# Characterization of LipS1 and LipS2 from *Thermococcus kodakarensis*: Proteins Annotated as Biotin Synthases, which Together Catalyze Formation of the Lipoyl Cofactor

**DOI:** 10.1021/acsbiomedchemau.2c00018

**Published:** 2022-07-14

**Authors:** Syam Sundar Neti, Debangsu Sil, Douglas M. Warui, Olga A. Esakova, Amy E. Solinski, Dante A. Serrano, Carsten Krebs, Squire J. Booker

**Affiliations:** ‡ Department of Chemistry, The Pennsylvania State University, University Park, Pennsylvania 16802, United States; § Department of Biochemistry and Molecular Biology, The Pennsylvania State University, University Park, Pennsylvania 16802, United States; # Howard Hughes Medical Institute, The Pennsylvania State University, University Park, Pennsylvania 16802, United States

**Keywords:** Lipoyl synthase, iron−sulfur clusters, radical SAM (RS); *S*-adenosylmethionine (SAM), biotin synthase, lipoic acid

## Abstract

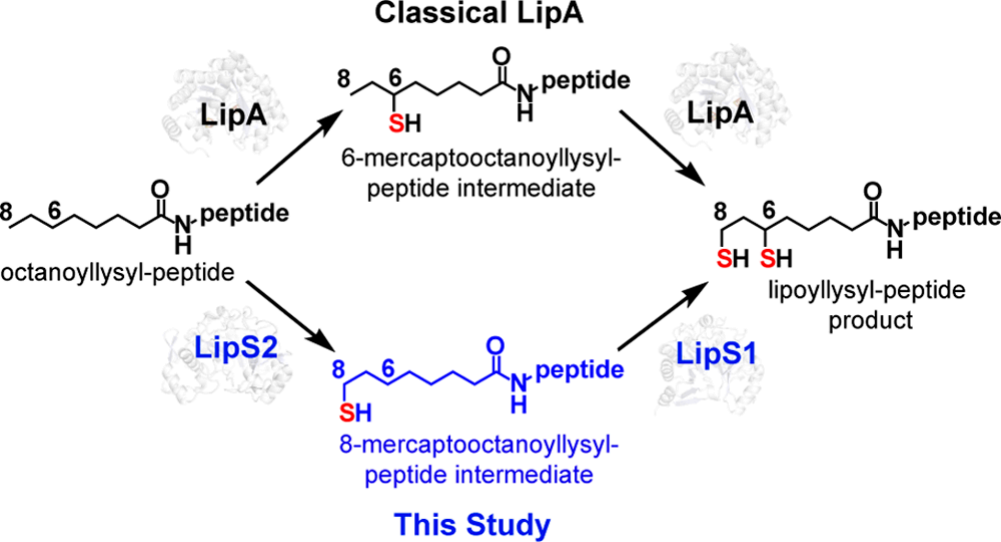

Lipoic acid is an eight-carbon sulfur-containing biomolecule that functions primarily as a cofactor in several multienzyme complexes. It is biosynthesized as an attachment to a specific lysyl residue on one of the subunits of these multienzyme complexes. In *Escherichia coli* and many other organisms, this biosynthetic pathway involves two dedicated proteins: octanoyltransferase (LipB) and lipoyl synthase (LipA). LipB transfers an *n*-octanoyl chain from the octanoyl-acyl carrier protein to the target lysyl residue, and then, LipA attaches two sulfur atoms (one at C6 and one at C8) to give the final lipoyl cofactor. All classical lipoyl synthases (LSs) are radical *S*-adenosylmethionine (SAM) enzymes, which use an [Fe_4_S_4_] cluster to reductively cleave SAM to generate a 5′-deoxyadenosyl 5′-radical. Classical LSs also contain a second [Fe_4_S_4_] cluster that serves as the source of both appended sulfur atoms. Recently, a novel pathway for generating the lipoyl cofactor was reported. This pathway replaces the canonical LS with two proteins, LipS1 and LipS2, which act together to catalyze formation of the lipoyl cofactor. In this work, we further characterize LipS1 and LipS2 biochemically and spectroscopically. Although LipS1 and LipS2 were previously annotated as biotin synthases, we show that both proteins, unlike *E. coli* biotin synthase, contain two [Fe_4_S_4_] clusters. We identify the cluster ligands to both iron–sulfur clusters in both proteins and show that LipS2 acts only on an octanoyl-containing substrate, while LipS1 acts only on an 8-mercaptooctanoyl-containing substrate. Therefore, similarly to *E. coli* biotin synthase and in contrast to *E. coli* LipA, sulfur attachment takes place initially at the terminal carbon (C8) and then at the C6 methylene carbon.

## Introduction

**Figure 1:**
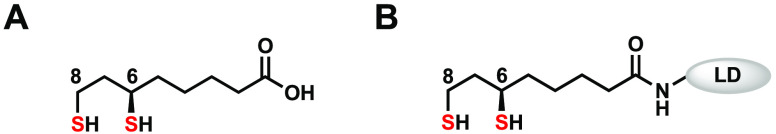
Chemical structures of (A) reduced lipoic acid and (B) reduced lipoyl cofactor (LD = lipoyl domain).

Lipoic acid (1,2-dithiolane-3-pentanoic acid or 6,8 thioctic acid) is a sulfur-containing biomolecule used as a central cofactor in several multienzyme complexes, including the pyruvate, α-ketoglutarate, 2-oxoacid, and 2-ketoadipate dehydrogenase complexes as well as in the glycine cleavage system (GCS).^1−3^ It consists of *n*-octanoic acid with sulfur atoms attached at C6 (*R* configuration) and C8 (Figure 1). In its cofactor form, it is attached in an amide linkage to a specific lysyl residue on lipoyl domains in subunits of these complexes. The resulting ∼14 Å appendage allows the cofactor to deliver intermediates into the active sites of other proteins in these complexes.^4^ Lipoic acid is biosynthesized directly in its cofactor form as an offshoot of type II fatty acid biosynthesis, which takes place on an acyl carrier protein (ACP). In *Escherichia coli* and many other organisms, octanoyltransferase (LipB) catalyzes the first committed step in the biosynthesis of the lipoyl cofactor, which is the transfer of the *n*-octanoyl chain from *n*-octanoyl-ACP to the H protein of the GCS or the E_2_ subunits of the pyruvate or α-ketoglutarate dehydrogenase complexes.^1,5−7^ In the second and last step, lipoyl synthase (LipA in *E. coli,* Lip5 in yeast, and LIAS in humans), attaches sulfur atoms at C6 and C8 of the octanoyl chain to yield the intact lipoyl cofactor.^5,8−10^

**Figure 2:**
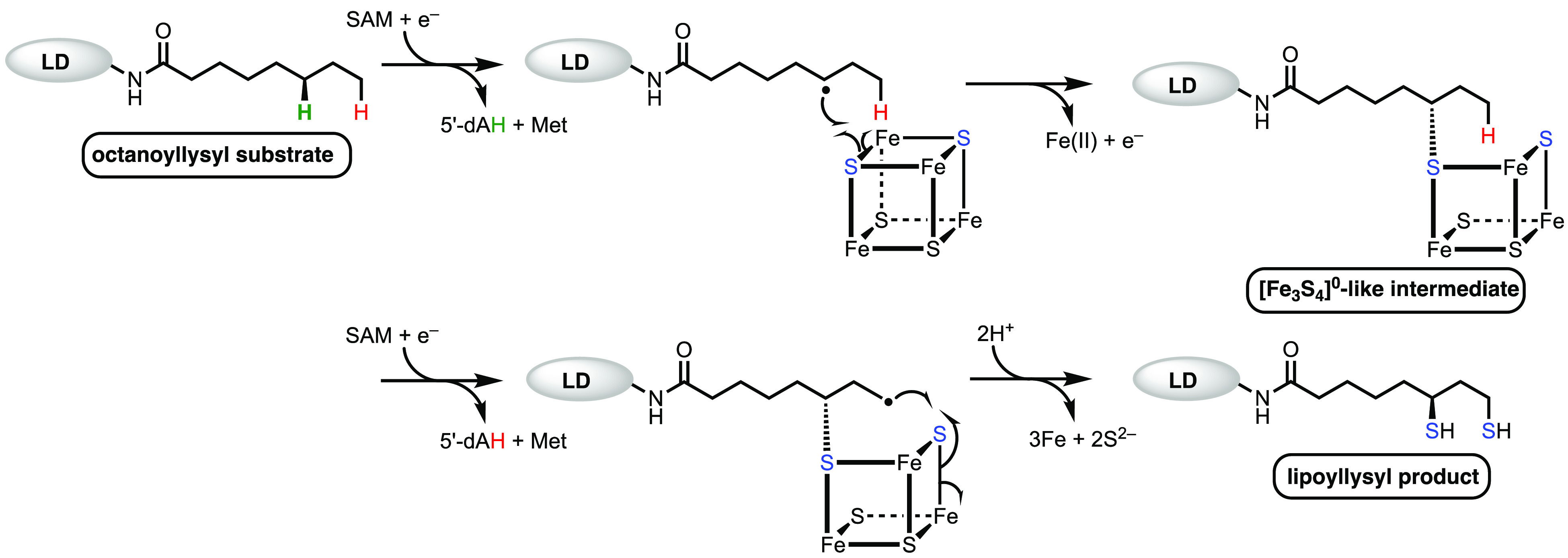
Proposed mechanism of the classical lipoyl synthase reaction.

LipA is a radical *S*-adenosylmethionine (SAM) enzyme, meaning that it uses SAM as a precursor to a 5′-deoxyadenosyl 5′-radical (5′-dA·).^10−14^ The 5′-dA·, a potent oxidant, removes hydrogens atoms (H·) from C6 and C8 of the octanoyl chain to allow for subsequent sulfur attachment through a mechanism involving organic radicals.^15^ All radical SAM (RS) enzymes contain at least one [Fe_4_S_4_] cluster ligated by cysteines most often residing in a Cx_3_Cx_2_C motif and required for the reductive cleavage of SAM.^16−20^ Lipoyl synthases contain a second cluster, termed the auxiliary cluster ([Fe_4_S_4_]_aux_), which is ligated by cysteines residing in an N-terminal Cx_4_Cx_5_C motif and the second serine in a highly conserved C-terminal RS**S**Y motif.^11,21,22^ The [Fe_4_S_4_]_aux_ is degraded during turnover to provide the sulfur atoms in the lipoyl product.^23^ The prevailing reaction mechanism for LipA catalysis is shown in Figure 2. One molecule of SAM binds to the RS cluster, [Fe_4_S_4_]_RS_, and is reductively cleaved to generate the 5′-dA·, which abstracts the C6 *pro-R* H· from the octanoyllysyl-containing substrate.^11−13,23,24^ The resulting substrate radical then attacks a bridging μ_3_-sulfido ion of the cluster with concomitant inner-sphere electron transfer to the cluster.^1,11,12^ Upon loss of an Fe^2+^ ion and an additional electron to an unknown acceptor, an intermediate is formed, in which C6 of the octanoyllysyl-containing substrate coordinates an [Fe_3_S_4_]^0^-like cluster, as was determined both by X-ray crystallography and Mössbauer spectroscopy.^11,12^ In the next step of the reaction, which is not as well characterized, a second molecule of SAM is cleaved to give a second 5′-dA·, which abstracts an H· from C8 of the octanoyllysyl-containing intermediate.^25,26^ The resulting substrate radical attacks a μ_2_-sulfido ion of the cluster with concomitant inner-sphere electron transfer to the cluster. The addition of two protons results in release of the lipoyl cofactor in its reduced form.^25−27^

Lipoyl synthases constitute one of several classes of RS enzymes that catalyze sulfur attachment to unactivated carbon centers.^27,28^ Others include biotin synthase (BioB), MiaB, MtaB, and RimO (Figure S1). BioB catalyzes the last step in the biosynthesis of biotin, which is the insertion of a sulfur atom between C6 and C9 of dethiobiotin.^29−31^ MiaB, MtaB, and RimO are methylthiotransferases.^32,33^ MiaB and its orthologues catalyze the attachment of a methylthio group (−SCH_3_) at C2 of *N*^6^-isopentenyladenosine 37 in select tRNAs, while MtaB and its orthologues catalyze the attachment of a methylthio group at C2 of *N*^6^-(threonylcarbamoyl)adenosine in select tRNAs.^34−36^ By contrast, RimO acts on a protein substrate, catalyzing the attachment of a methythio group at C3 of Asp89 on protein S12 of the bacterial ribosome in many organisms.^37−41^ Like LipA, these enzymes also contain auxiliary clusters that appear to be sacrificed during turnover. BioB contains an [Fe_2_S_2_]_aux_ cluster,^42,43^ while MiaB, MtaB, and RimO contain [Fe_4_S_4_]_aux_ clusters.^32^

Recently, it was reported that the genome of *Thermococcus kodakarensis* does not encode a canonical lipoyl synthase but instead uses two gene products in tandem (LipS1 and LipS2) to perform each of the sulfur attachment steps.^44^ Interestingly, LipS1 and LipS2 were annotated as biotin synthases, but the isolated proteins, when together, were shown to generate a lipoyl group on an octanoyllysyl-containing peptide substrate surrogate. In *T. kodakarensis*, LipS1 and LipS2 are not clustered; they are >130 genes apart on the chromosome. However, in many other organisms, they appear to be in the same genome neighborhood (within 10 genes) and are often located adjacent to each other. Herein, we isolate and characterize LipS1 and LipS2. We show that, in contrast to *E. coli* BioB, both proteins contain [Fe_4_S_4_] rather than [Fe_2_S_2_] auxiliary clusters. We further show that similarly to the *E. coli* BioB reaction and in stark contrast to the canonical LipA reaction, LipS2 catalyzes the initial sulfur attachment first at the terminal carbon (C8), while LipS1 catalyzes subsequent sulfur attachment at the C6 methylene carbon. We also identify ligands to each of the two clusters in both proteins.

## Materials and Methods

### Materials

*N*-(2-Hydroxyethyl)-piperazine-*N*′-(2-ethanesulfonic acid) (HEPES) was purchased from Fisher Scientific. Imidazole was purchased from J. T. Baker Chemical Co. Potassium chloride and glycerol were purchased from EMD Chemicals. β-Mercaptoethanol (BME), sodium dithionite, sodium sulfide, 5′-deoxyadenosine (5′-dAH), and *S*-adenosylhomocysteine (SAH) were purchased from MilliporeSigma. Kanamycin, ampicillin, dithiothreitol (DTT), arabinose, isopropyl β-d-1-thiogalactopyranoside (IPTG) and tris(2-carboxyethyl)phosphine hydrochloride (TCEP) were purchased from Gold Biotechnology. Ni-nitrilotriacetic acid (NTA) resin was acquired from Qiagen. SAM was synthesized and purified as described previously.^45^ DNA isolation kits were purchased from Macherey-Nagel (Dueren, Germany). All other chemicals and materials were of the highest grade available and were from MilliporeSigma.

### Synthesis of Peptide Substrates

All peptides were custom synthesized by Proimmune (Oxford, UK). AtsA peptide (Pro-Met-Ser-Ala-Pro-Ala-Arg-Ser-Met) was used as an external standard for quantification of peptides during liquid chromatography−mass spectrometry (LC-MS) analysis.^46^ The sequences of peptides used in this study are as follows: octanoyllysyl peptide (Glu-Ser-Val-(*N*^6^-octanoyl)Lys-Ala-Ala-Ser-Asp) (**peptide 1**), 6-mercaptooctanoyllysyl peptide (**peptide 2**), 8-mercaptooctanoyllysyl peptide (**peptide 3**), and lipoyl peptide (**peptide 4**).

### General Methods

UV–visible spectra were recorded on a Varian Cary 50 spectrometer (Agilent, Walnut Creek, CA) using the WinUV software package to control the instrument. High-performance liquid chromatography (HPLC) with detection by tandem mass spectrometry (LC-MS/MS) was conducted on an Agilent Technologies 1200 system coupled to an Agilent Technologies 6410 QQQ mass spectrometer. The system was operated with the associated MassHunter software package, which was also used for data collection and analysis. HPLC was also conducted on an Agilent 1100 Series system coupled to an Agilent 1100 Series variable wavelength detector and quaternary pump.

### Plasmids and Strains

Genes encoding *Thermococcus kodakarensis* LipS1 (TK2109) and LipS2 (TK2248) were codon-optimized using GeneArt software (Thermo Fisher) for expression in *E. coli*. The genes were subcloned into pET-28a(+) vectors using *Nde*I and *Xho*I restriction sites. The resulting plasmids, pET-28a(+)-LipS1 and pET-28a(+)-LipS2, were used to transform *E. coli* BL21 (DE3) competent cells containing the pDB1282 plasmid, which harbors the *isc* operon from *Azotobacter vinelandii*.^47^ Site-directed mutagenesis was performed using pET-28a(+)-LipS1 and pET-28a(+)-LipS2 plasmids as templates along with primers shown in Tables S1–S3. The resulting sequence changes were verified by DNA sequencing at the Penn State Genomics Core Facility (University Park, PA), and the resulting plasmids were used to transform *E. coli* BL21 (DE3) competent cells. Primers used for site-directed mutagenesis were purchased from Integrated DNA Technologies (Coralville, IA).

### Overproduction of Wild Type TK2109 (LipS1) or Wild Type TK2248 (LipS2)

A 200 mL starter culture containing 50 μg/mL kanamycin and 100 μg/mL ampicillin was inoculated with a single colony and incubated at 37 °C for 12 h with shaking (180 rpm). A 10 mL aliquot of the starter culture was used to inoculate 16 L of LB medium containing 50 μg/mL kanamycin and 100 μg/L ampicillin and incubated at 37 °C with shaking (180 rpm) until an optical density at 600 nm (OD_600_) of 0.3 was reached. At an OD_600_ ≈ 0.3, 50 μM FeCl_3_ and 50 μM l-cysteine were added while 0.2% (w/v) l-arabinose was also added to induce the expression of genes on the pDB1282 plasmid. At an OD_600_ ≈ 0.6, LipS1 or LipS2 expression was induced by adding 0.25 mM IPTG, and incubation was continued at 37 °C with shaking (180 rpm) for an additional 12 h. The cells were harvested by centrifugation (4 °C, 6000*g*, 15 min), flash-frozen in liquid N_2_, and stored at −80 °C until use.

### Purification of LipS1 or LipS2 and Corresponding Variants of Each Protein

The purification of WT LipS1 or LipS2, as well as corresponding variants, was performed in an anaerobic chamber containing <1 ppm of O_2_ (Coy Laboratory products, Grass Lake, Michigan). Cells were resuspended in 200 mL of lysis buffer (100 mM Tris-HCl, pH 8.0, 150 mM KCl, 10 mM imidazole, 10 mM BME, 10 mM MgCl_2_, 0.25 mM FeCl_3_, 1 mM l-cysteine, and 1 mM pyridoxal 5′-phosphate (PLP)) and disrupted by sonication with an ultrasonic cell disruptor (Branson Sonifier II “Model W- 250”, Heinemann). The lysates were then clarified by centrifugation (4 °C, 45 000*g*, 1 h). The N-terminally His_6_-tagged LipS1 or LipS2 was then purified by Ni-NTA affinity chromatography. The Ni-NTA resin was equilibrated with 100 mL of lysis buffer. After the supernatant was loaded onto the Ni-NTA column, the resin was washed with 200 mL of wash buffer (50 mM HEPES, pH 7.5, 300 mM KCl, 30 mM imidazole, 10% glycerol (v/v), 10 mM BME, and 0.5 mM DTT). Protein elution from the Ni-NTA resin was performed with 30–35 mL of elution buffer (50 mM HEPES, pH 7.5, 250 mM KCl, 300 mM imidazole, 10% glycerol, 10 mM BME, and 0.5 mM DTT). The protein was concentrated (Millipore, 10 kDa molecular weight cutoff (MWCO)), and imidazole was removed using a PD-10 column (GE Healthcare). The proteins were then subjected to chemical reconstitution with FeCl_3_ and Na_2_S as previously described and purified further by size-exclusion chromatography on a HiPrep 16/60 Sephacryl HR S-200 column (Cytiva) equilibrated in 50 mM HEPES, pH 7.5, 250 mM KCl, 15% glycerol, and 10 mM DTT at a flow rate of 0.5 mL/min.^47^ The S-200 column was connected to an AKTA protein liquid chromatography system (Cytiva) located in an anaerobic chamber. Fractions containing the target protein were combined and concentrated using centricons (Millipore, 10 kDa MWCO), and the final enzyme solution was flash-frozen in liquid N_2_ and stored under liquid N_2_ until use. Protein homogeneity was judged by sodium dodecyl sulfate–polyacrylamide gel electrophoresis (SDS-PAGE), and protein concentration was determined by the method of Bradford, using bovine serum albumin (fraction V) as a standard.^48^ Amino acid analysis was performed by the UC Davis proteomics core facility as previously described and revealed that the Bradford method overestimates LipS1 concentration by a factor of 1.5^47^ By contrast, no correction factor is needed for LipS2. Colorimetric iron and sulfide analyses were conducted on the purified protein using the methods of Beinert.^49−51^

### Overproduction and Purification of ^57^Fe-Labeled Proteins for Mössbauer Spectroscopy

To generate ^57^Fe-labeled proteins for analysis by Mössbauer spectroscopy, LipS1, LipS2, or LipS1 and LipS2 variants containing three simultaneous Cys → Ala substitutions in the corresponding Cx_3_Cx_2_C motifs (i.e., Ax_3_Ax_2_A variants, also denoted as LipS1_ΔRS_, LipS2_ΔRS_), were overproduced in *E. coli* cultured in M9 minimal media supplemented with 50 μM ^57^FeCl_3_. The growth and purification procedures were essentially identical to those described above, with the exception that ^57^FeCl_3_ was also used for chemical reconstitution. ^57^FeCl_3_ was prepared as previously described.^52^ For analysis by Mossbauer spectroscopy, 300 μM purified and ^57^Fe-labeled LipS1 or LipS2 were loaded into Mössbauer cups and flash-frozen in liquid nitrogen before data collection. For LipS1_ΔRS_ and LipS2_ΔRS_, respectively, ∼650 μM of each protein was used.

Mossbauer spectra were recorded on a spectrometer from SEECO (Edina, MN) equipped with a Janis SVT-400 variable-temperature cryostat. The reported isomer shifts are given relative to the centroid of the spectrum of an α-iron metal at room temperature. External magnetic fields were applied parallel to the direction of propagation of the γ radiation. Simulations of Mossbauer spectra were carried out using the WMOSS spectral analysis software from SEECO (www.wmoss.org, SEE Co., Edina, MN).

### LC-MS Activity Assays

Activity measurements were conducted in a Coy anaerobic chamber containing <1 ppm of O_2_. Each reaction contained the following at their final concentrations in a final volume of 200 μL: 50 mM HEPES, pH 7.5, 50 mM KCl, 10 μM LipS1 and/or 10 μM LipS2, 0.5 mM SAM, 100 μM L-tryptophan (internal standard for 5′-dAH), and 300 μM peptide substrate (**peptide 1**, **peptide 2**, or **peptide 3**). Reactions were initiated by the addition of 1 mM (final concentration) sodium dithionite, and 25 μL aliquots were removed at various times and added to an equal volume of 300 mM H_2_SO_4_ containing 10 μM AtsA peptide (external standard for peptide substrates) and 8 mM TCEP. Assays using the ferredoxin/ferredoxin reductase/NADPH reducing system contained 10 μM ferredoxin, 10 μM ferredoxin reductase, and 1 mM NADPH.^53^ All assay components were pre-incubated for 10 min in the absence of peptide substrate. Reactions were initiated by the addition of the peptide substrate, and 25 μL aliquots were removed at appropriate times and mixed with a quench solution as described above. The samples were centrifuged at 14 000*g* for 30 min and analyzed by LC-MS using multiple-reaction monitoring (MRM). The time-dependent formation of lipoyl peptide product, monothiolated intermediate, and 5′-dAH as well as the decay of the octanoyllysyl-containing peptide substrate (**peptide 1**) or 8-mercaptooctanoyllysyl peptide substrate (**peptide 3**) were determined by LC-MS/MS with MRM using the conditions described in Tables S4 and S5. Detection of substrates and products was performed using electrospray ionization (ESI^+^) in positive mode, and quantification was based on standard curves of substrates and products. The assay mixture was separated on an Agilent Technologies Zorbax Extend-C18 column Rapid Resolution HT (4.6 × 50 mm, 1.8 μm particle size) equilibrated in 95% Solvent A (0.1% formic acid, pH 2.6) and 5% Solvent B (100% acetonitrile). A gradient of 5–50% Solvent B was applied from 0.5 to 3 min and maintained at 50% Solvent B for 4.5 min before returning to 5% Solvent B from 4.5 to 5.5 min. A flow rate of 0.3 mL/min was maintained throughout the method. The column was allowed to re-equilibrate for an additional 1.5 min under the initial conditions between sample injections.

### Electron Paramagnetic Resonance Spectroscopy Analysis of LipS1

Three samples of LipS1 were prepared in an anaerobic chamber for analysis by electron paramagnetic resonance (EPR) spectroscopy. The samples contained 200 μM LipS1_WT_, 200 μM LipS1_WT_ + 2 mM dithionite, or 200 μM LipS1_WT_ + 2 mM dithionite + 1 mM SAM in a final volume of 300 μL. Samples containing dithionite were reduced for 15 min with freshly prepared dithionite and flash-frozen in cryogenic isopentane. Continuous-wave EPR spectra were collected at 10 K with a microwave power of 10 mW and a modulation amplitude of 0.2 mT on a Magnettech 5000 X-band ESR spectrometer (Bruker) equipped with an ER 4102ST resonator. Temperature was controlled by an ER 4112-HV (Oxford Instruments, Concord MA) variable-temperature helium-151 flow cryostat.

### Overexpression and Purification of ^34^S-Labeled LipS1 and LipS2

The expression of apo-LipS1 and LipS2 was performed as described for the expression of *E. coli* NfuA with the exception that no FeCl_3_ or cysteine was added during the cell growth or lysis.^23^ Purification and chemical reconstitution of apo-LipS1 and apo-LipS2 with FeCl_3_ and Na_2_^34^S was performed as described above. The reconstituted LipS1 and LipS2 proteins were centrifuged at 14 000*g* for 10 min to remove aggregates and further purified by size-exclusion chromatography as described above. The synthesis of Na_2_^34^S was performed as previously reported.^23^

### Generation of GNNs

The genome neighborhood networks (GNNs) of LipS1 and LipS2 (TK2109 and TK2248) from *Thermococcus kodakarensis* were constructed using information available on radicalSAM.org with further processing in Cytoscape 3.9.1.^54^ TK2109 (Q5JEV3) was found in megacluster 4–7 (https://radicalsam.org/explore.php?id=cluster-4-7&v=3.0), and TK2248 (Q5JH59) was found in megacluster 4–8 (https://radicalsam.org/explore.php?id=cluster-4-8&v=3.0). Megacluster 4–7 (LipS1) and 4–8 (LipS2) were downloaded and opened in Cytoscape (accessed March 2022). All visualization used the yFiles organic layout. A node column filter (Node: superkingdom) was used to split each megacluster into two subclusters, resulting in four total subclusters: 4–7_bacteria, 4–7_archaea, 4–8_bacteria, and 4–8_archaea. All four subclusters were submitted to the genome neighborhood tool (GNT) on enzymefunction.org.^55,56^ The search included standard settings, including a neighborhood size of 10 and a minimal co-occurrence percentage lower limit of 20%. The GNNs were downloaded, and the resultant Pfam Family Hub-Nodes were opened in Cytoscape for analysis.

## Results

Recently, Atomi and co-workers showed that both LipS1 (TK2109) and LipS2 (TK2248) from the hyperthermophilic archaeon *Thermococcus kodakarensis* are involved in lipoic acid biosynthesis.^44^ This organism does not contain a gene encoding a classical LS but contains genes that were annotated as encoding biotin synthases. They also showed, using a chemically synthesized octanoyllysine-containing octapeptide corresponding to a sequence of the H protein of the GCS, that the recombinant proteins, only when together, catalyze the formation of the lipoyl group, suggesting that one protein attaches sulfur at C8, while the second attaches sulfur at C6. However, several questions remain, such as how similar is each of these proteins to the classical BioB and what is the nature of the sulfur donors in the reaction? Inspired by these questions, a detailed characterization of each of the two enzymes was initiated.

**Figure 3:**
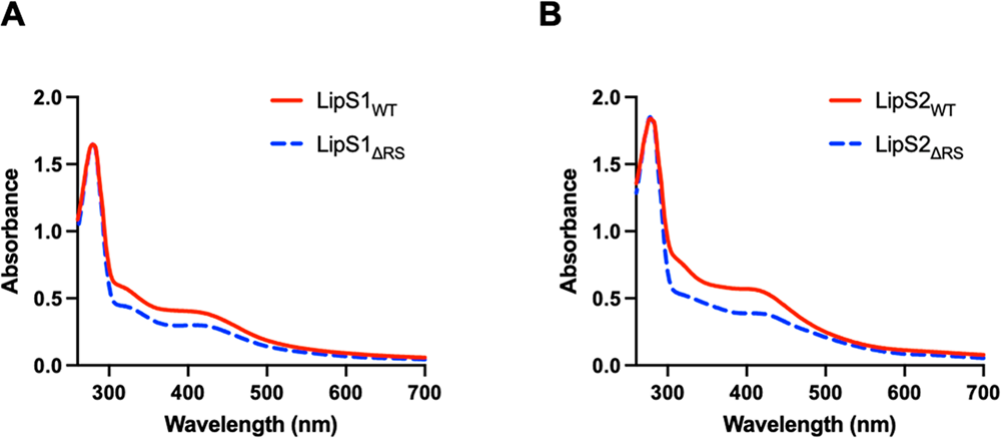
UV–vis spectra of WT LipS1 and LipS2 proteins and their triple-variants (normalized to the absorption at 280 nm). (A) LipS1_WT_ (20 μM) and LipS1_ΔRS_ (20 μM). (B) LipS2_WT_ (20 μM) and LipS2_ΔRS_ (20 μM).

Genes encoding LipS1 and LipS2 were engineered to produce proteins containing an N-terminal hexahistidine (His_6_) tag that is separated from the first amino acid of the authentic protein by a spacer of 10 amino acids. Protein overproduction was conducted in the presence of plasmid pDB1282, which contains genes from *Azotobacter vinelandii* that encode proteins involved in iron–sulfur (Fe/S) cluster biosynthesis.^47,57^ Each of the proteins was then isolated by immobilized metal affinity chromatography (IMAC) and then subjected to amino acid analysis to determine a correction factor for the Bradford protein assay using BSA (fraction V) as a standard. It was observed that the Bradford method overestimates the concentration of LipS1 by a factor of 1.5, while no correction factor is needed for LipS2. The UV–vis spectra of purified LipS1 (Figure 3A, red line) and LipS2 (Figure 3B, red line) are consistent with the expected presence of [Fe_4_S_4_] clusters, although UV–vis spectroscopy is not a robust indicator of cluster types. Iron and sulfide analyses coupled with protein concentration determination indicate that reconstituted LipS1 contains 6.6 ± 0.3 irons and 10.4 ± 0.1 sulfides per polypeptide, and reconstituted LipS2 contains 7.1 ± 0.4 irons and 6.2 ± 0.1 sulfides per polypeptide.

Given the expectation that both LipS1 and LipS2 contain auxiliary Fe/S clusters as well as that the iron and sulfide content exceeds four per polypeptide for each protein, we generated a triple-variant of each of the proteins in which all three cysteines in the Cx_3_Cx_2_C motif (Cys 27, 31, and 34 for LipS1 (called LipS1_ΔRS_) and Cys 39, 43, and 46 for LipS2 (called LipS2_ΔRS_)) were changed to alanine. The expectation is that the [Fe_4_S_4_]_RS_ cluster will be deleted, allowing the auxiliary cluster to be isolated and characterized. Reconstituted LipS1_ΔRS_ contains 4.1 ± 0.1 irons and 5.0 ± 0.1 sulfides per polypeptide, while reconstituted LipS2_ΔRS_ contains 3.9 ± 0.1 irons and 4.2 ± 0.1 sulfides per polypeptide. The UV–vis spectra are displayed in Figure 3 (blue dashed lines). As can be observed, the overall spectral envelope does not change significantly for either protein and is consistent with remaining [Fe_4_S_4_] clusters.

### Mössbauer-Spectroscopic Evidence for Auxiliary [Fe_4_S_4_] Clusters in LipS1 and LipS2

**Figure 4:**
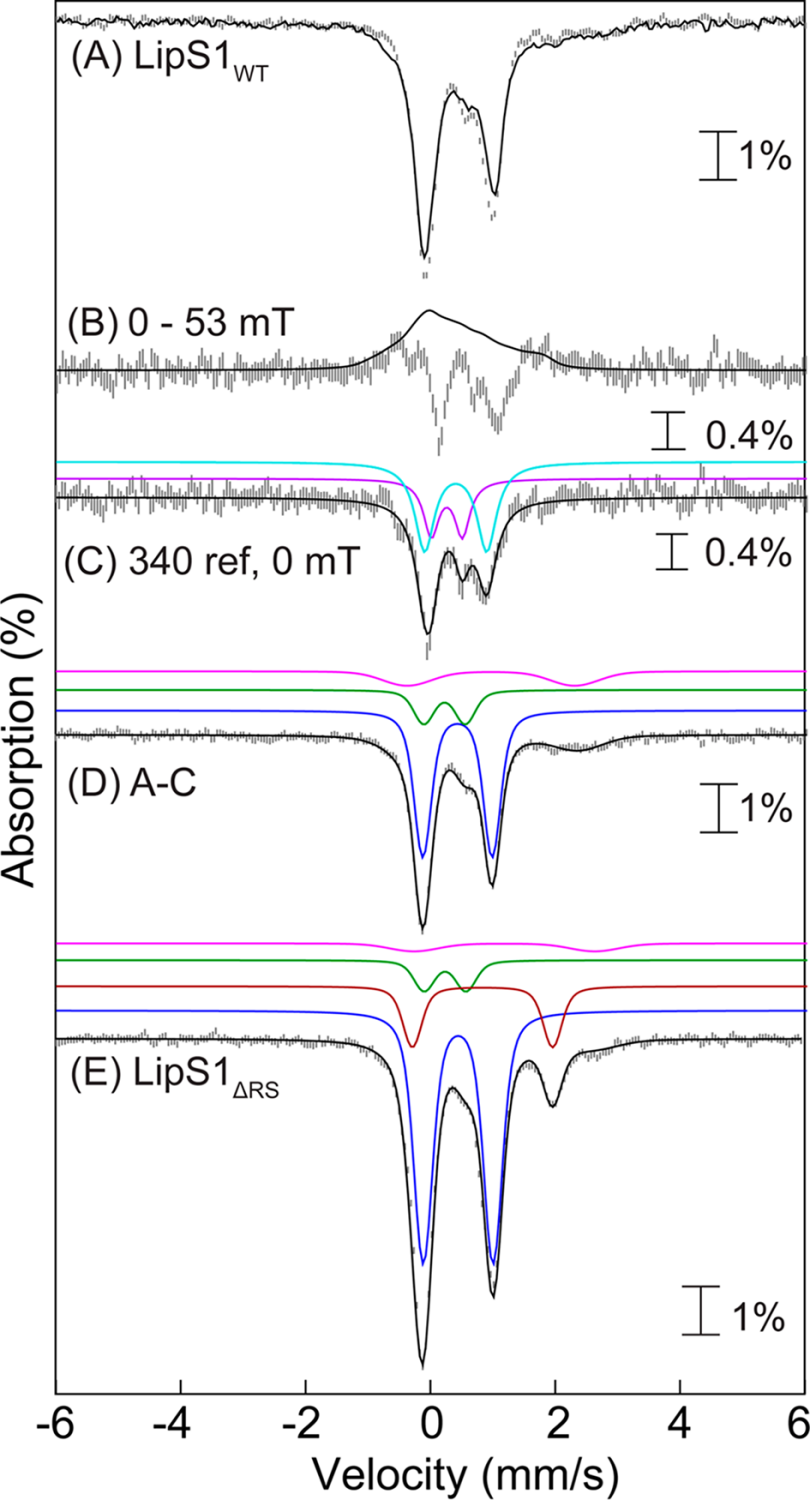
(A) Mössbauer spectra of reconstituted LipS1_WT_ collected at 4.2 K with an external applied magnetic field of either 0 mT (vertical bars) or 53 mT oriented parallel to the γ beam (solid line). (B) [0–53 mT] difference spectrum (vertical bars) and simulation of the LipS1_WT_ [Fe_3_S_4_]^0^ cluster in 53 mT using previously reported parameters^12^ (black line). (C) Zero-field reference spectrum of the LipS1_WT_ [Fe_3_S_4_]^0^ cluster (vertical bars). Black line is the simulation of the LipS1_WT_ [Fe_3_S_4_]^0^ cluster in zero field with parameters from Table S6. Individual contributions from the exchange coupled Fe_2_^2.5^ (cyan line) and Fe^III^ (purple line) sites with a 2:1 intensity ratio. (D) 0 mT – 30% zero-field reference spectrum of the LipS1_WT_ [Fe_3_S_4_]^0^ cluster (vertical bars) showing the presence of three quadrupole doublets corresponding to [Fe_4_S_4_]^2+^ (47% of total intensity, blue line), [Fe_2_S_2_]^2+^ (12% of total intensity, green line) clusters, and N/O-coordinated high-spin Fe^II^ (11% of total intensity, pink line). The black line represents the overall simulated spectrum. (E) Mössbauer spectra of reconstituted LipS1_ΔRS_ collected at 4.2 K in absence of any externally applied magnetic field. The black line represents the overall simulated spectrum. The blue and brown lines are individual contributions from the [Fe_4_S_4_]^2+^ cluster (69% of the total intensity) and the unique Fe site of a [Fe_4_S_4_]^2+^ cluster coordinated by 2–3 N/O ligands (17% of the total intensity), respectively. The green and pink lines show the contributions from [Fe_2_S_2_]^2+^ clusters (9% of total intensity) and N/O-coordinated high-spin Fe^II^ (5% of total intensity), respectively.

To determine the type and stoichiometry of Fe/S clusters associated with LipS1 and LipS2, Mössbauer and EPR spectroscopies were used to analyze samples of each ^57^Fe-enriched protein. The 4.2 K Mössbauer spectra of wild type LipS1 (LipS1_WT_) collected in an external magnetic field of 0 or 53 mT (Figure 4A) are dominated by the two prominent lines of a quadrupole doublet with parameters typical of [Fe_4_S_4_]^2+^ clusters (isomer shift (δ) of 0.46 mm/s and quadrupole splitting parameter (Δ*E*_Q_) of 1.12, 47% of total intensity, blue lines).^58^ The spectra also exhibit the typical field-dependence associated with [Fe_3_S_4_]^0^ clusters (Figure 4B), in particular, the 2:1 intensity ratio of quadrupole doublets attributable to the Fe_2_^2.5^ and Fe^III^ sites (Figure 4C, cyan and purple lines, see Table S6 for parameters, 30% of total intensity).^12^ Further analysis of the spectra reveals small amounts of [Fe_2_S_2_]^2+^ clusters (12% of total intensity, Figure 4D) and N/O-coordinated high-spin Fe^II^ (11% of total intensity). The EPR spectrum of this sample (Figure S2) exhibits a weak signal typical of [Fe_3_S_4_]^+^ clusters, which is below the detection limit of Mössbauer spectroscopy (∼1–2% of total Fe). Combined with the LipS1_WT_ stoichiometry of 7.0 irons, these results demonstrate the presence of 0.8 [Fe_4_S_4_], 0.7 [Fe_3_S_4_], and 0.4 [Fe_2_S_2_] clusters per LipS1. The results suggest that LipS1 contains two [Fe_4_S_4_] cluster sites, with [Fe_3_S_4_] and [Fe_2_S_2_] clusters presumably resulting from degradation or incomplete reconstitution of [Fe_4_S_4_] clusters. To provide stronger evidence for this assignment, we also characterized LipS1_ΔRS_ by Mössbauer spectroscopy. The 4.2 K Mössbauer spectrum of LipS1_ΔRS_ (Figures 4E and S3) is dominated by a quadrupole doublet with parameters identical to those of the [Fe_4_S_4_]^2+^ cluster of LipS1_WT_ (blue line, 69% of total intensity) and a weaker line at ∼+2 mm/s, which can be attributed to a quadrupole doublet with parameters (δ = 0.85 mm/s and Δ*E*_Q_ = 2.25 mm/s, brown line, Figure 4A) that are consistent with those of the unique Fe site of an [Fe_4_S_4_]^2+^ cluster coordinated by 2–3 N/O ligands.^12,16,59,60^ The 4.2 K Mössbauer spectrum of reconstituted LipS1_ΔRS_ is very similar to that of a sample of anoxically isolated LipS1_ΔRS_ that was not further reconstituted with ^57^FeCl_3_ and Na_2_S (Figure S3), thus suggesting that the presence of the [Fe_4_S_4_]^2+^ cluster is not an artifact due to the reconstitution procedure and that the auxiliary cluster of LipS1 is a [Fe_4_S_4_] cluster. Thus, the data reveal that the auxiliary binding site harbors an [Fe_4_S_4_] cluster. The presence of [Fe_2_S_2_]^2+^ and [Fe_3_S_4_]^0^ in the sample of LipS1_WT_ is therefore attributed to degradation of [Fe_4_S_4_]^2+^ clusters, as has been observed previously.^61,62^

**Figure 5:**
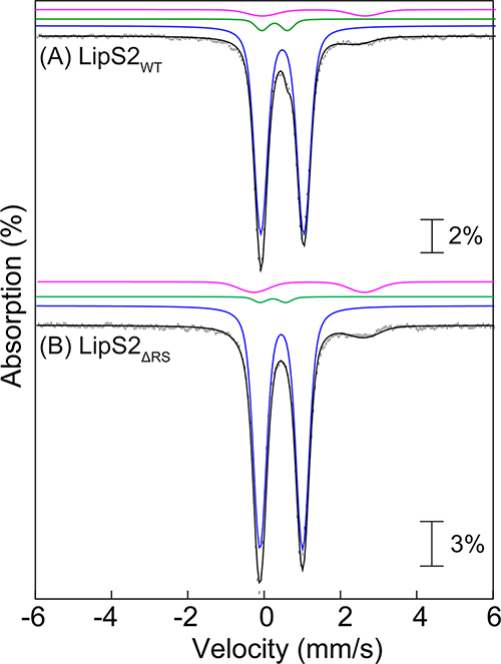
Mössbauer spectra of reconstituted (A) LipS2_WT_ and (B) LipS2_ΔRS_ collected at 4.2 K (vertical bars) in the absence of any external applied magnetic field. The black lines represent the overall simulated spectra, while the individual contributions from the [Fe_4_S_4_]^2+^ (90% of total intensity in both LipS2_WT_ and LipS2_ΔRS_), [Fe_2_S_2_]^2+^ (5 and 2% of total intensities in LipS2_WT_ and LipS2_ΔRS_, respectively) clusters, and N/O-coordinated high-spin Fe^II^ (5 and 8% of total intensities in LipS2_WT_ and LipS2_ΔRS_, respectively) are shown by the blue, green, and pink lines, respectively.

The 4.2 K Mössbauer spectra of LipS2_WT_ (Figures 5A and S4) are dominated by a quadrupole doublet with parameters suggestive of [Fe_4_S_4_]^2+^ clusters (δ = 0.47 mm/s and Δ*E*_Q_ = 1.12 mm/s, 90% of total intensity, blue line) in addition to smaller amounts of [Fe_2_S_2_]^2+^ clusters and N/O-coordinated Fe^II^ (5% of total intensity each). Together with the Fe/LipS2_WT_ stoichiometry of 6.8, the spectrum reveals the presence of 1.5 [Fe_4_S_4_] clusters per protein, which suggests that the protein contains two [Fe_4_S_4_] cluster binding sites. The 4.2 K Mössbauer spectrum of LipS2_ΔRS_ (Figures 5B and S5) is dominated by the quadrupole doublet associated with [Fe_4_S_4_]^2+^ clusters (90% of total intensity) and corresponds to 0.9 [Fe_4_S_4_] clusters per LipS2_ΔRS_. Thus, like LipS1, LipS2 also harbors two [Fe_4_S_4_] clusters.

### LipS1 and LipS2 Activity Determinations

**Figure 6:**
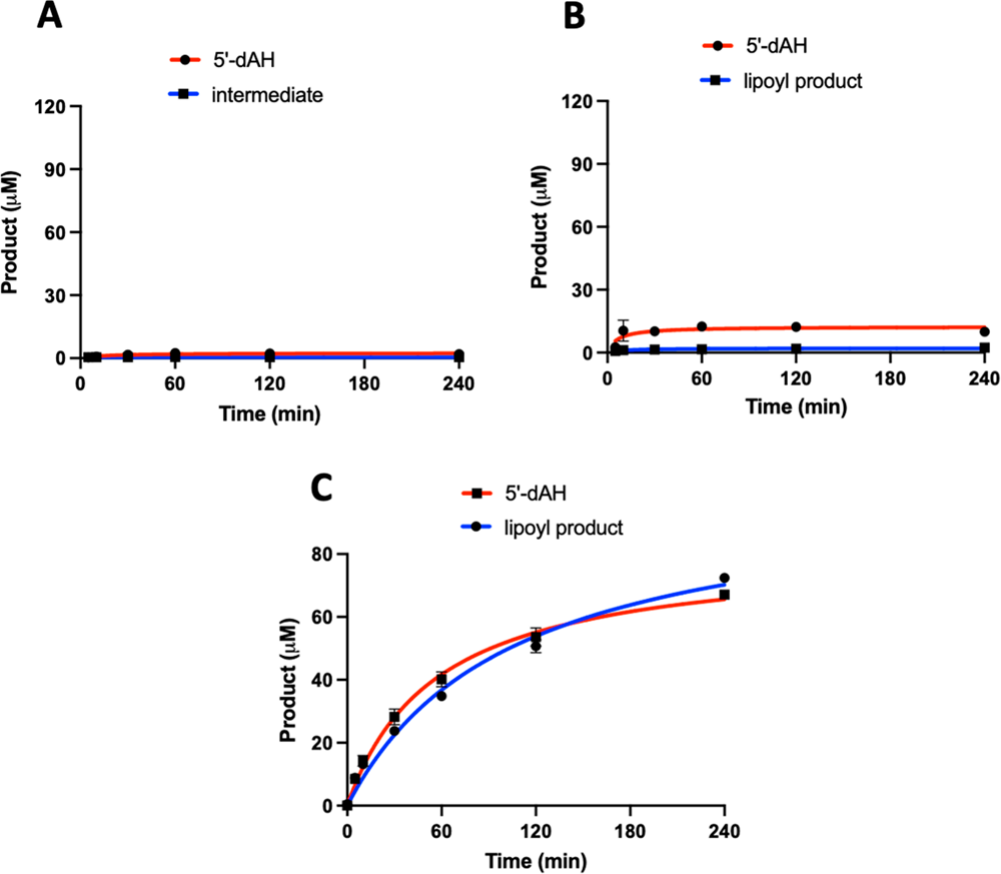
LipS1 attaches sulfur to an 8-mercaptooctanoyllysyl peptide only. The LipS1 reaction with (A) octanoyllysyl peptide (**peptide 1**), (B) 6-mercaptooctanoyllysyl peptide (**peptide 2**), and (C) 8-mercaptooctanoyllysyl peptide (**peptide 3**). The reactions contained 10 μM LipS1, 300 μM respective substrate, 1 mM dithionite, and 0.5 mM SAM. Reactions were conducted at 45 °C in triplicate. Error bars represent one standard deviation from the mean.

**Figure 7:**
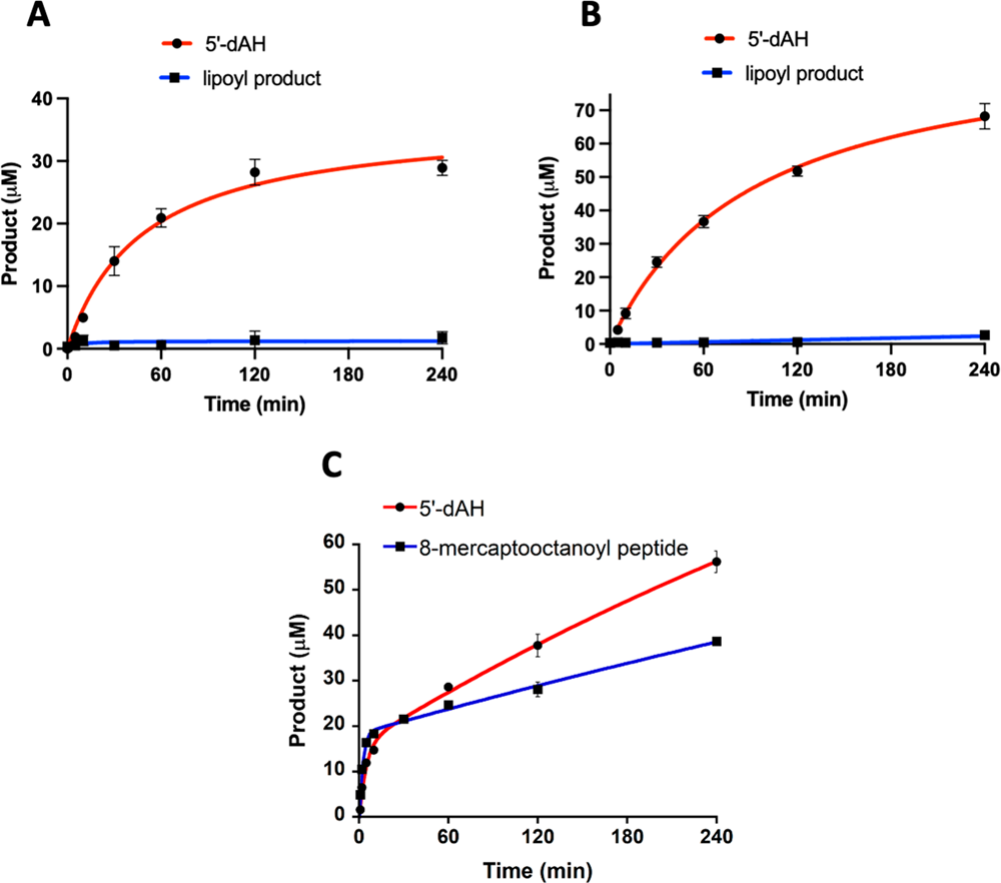
LipS2 attaches sulfur to an octanoyllysyl peptide only. The LipS2 reaction with (A) 6-mercaptooctanoyllysyl peptide, (B) 8-mercaptooctanoyllysyl peptide, and (C) octanoyllysyl peptide. The reactions contained 10 μM LipS2, 300 μM respective substrate, 1 mM dithionite, and 0.5 mM SAM. Reactions were conducted at 45 °C in triplicate. Error bars represent one standard deviation from the mean.

**Figure 8:**
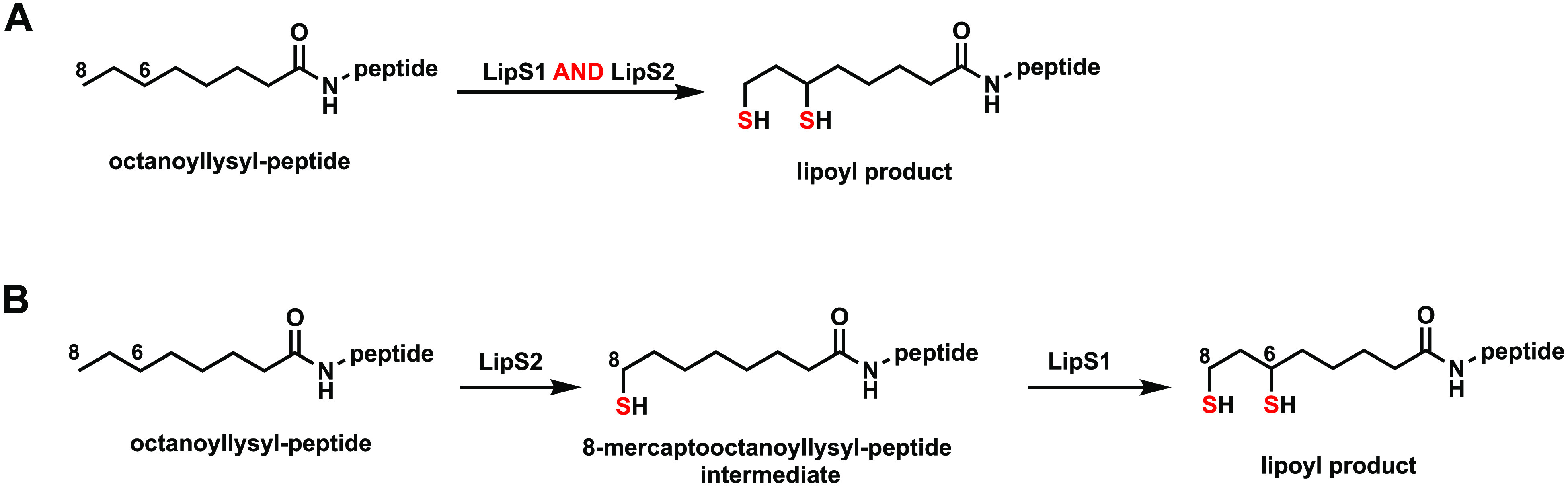
LipS1 and LipS2 reaction sequence. (A) Overall catalysis by LipS1 and LipS2. (B) Substrate preference and reaction sequence of LipS1 and LipS2.

LipS1 was tested for its ability to attach sulfur atoms to a peptide substrate surrogate consisting of Glu-Ser-Val-(*N*^6^-octanoyl)Lys-Ala-Ala-Ser-Asp (**peptide 1**) or **peptide 1** in which the octanoyllysyl residue is replaced with a 6-mercaptooctanoyllysyl (**peptide 2**) or 8-mercaptooctanoyllysyl group (**peptide 3**). When LipS1 (10 μM) is incubated with 500 μM SAM and 300 μM **peptide 1**, no sulfur-containing product is observed, although 2 μM 5′-deoxyadenosine (5′-dAH) is produced over 240 min (Figure 6A). A similar result is obtained when LipS1 is incubated with **peptide 2** under turnover conditions, although more than 10 μM 5′-dAH is formed (Figure 6B). By contrast, when LipS1 is incubated with **peptide 3**, formation of the lipoyl product (**peptide 4**) is observed, with a near 1:1 stoichiometry with 5′-dAH (Figure 6C). Interestingly, the amount of product obtained (∼70 μM) is significantly greater than the concentration of enzyme in the reaction (10 μM). As detailed previously, LipS1 contains ∼10 sulfides per polypeptide, suggesting that the enzyme can mobilize all four sulfurs of the auxiliary cluster or is able to obtain sulfide from extraneous sources (*vide infra*). A similar set of experiments was conducted with LipS2. LipS2 uses neither a 6-mercaptooctanoyl peptide substrate (**peptide 2**) nor an 8-mercaptooctanoyl peptide substrate (**peptide 3**) (Figure 7A,B, respectively), although these substrates induce abortive cleavage of SAM to generate 5′-dAH. However, LipS2 readily reacts with the octanoyllysyl peptide substrate (**peptide 1**), converting it into an 8-mercaptooctanoyl group (Figure 7C). This assignment is based on the observation that further reaction with LipS1 results in formation of the lipoyl group (Figure 6C). The kinetics of product formation indicates a burst of 2 equiv of the 8-mercaptooctanoyl species followed by a slower production of up to ∼4 equiv over 240 min. Based on these results, the reaction sequence of lipoyl product formation is sulfur attachment first at C8, catalyzed by LipS2, and then sulfur attachment at C6, catalyzed by LipS1 (Figure 8). This order of reactivity is in direct contrast to that of canonical LSs, wherein sulfur attachment occurs first at C6 and then at C8.^11,12,25^ However, it is analogous to that of the BioB reaction, wherein sulfur attachment occurs first at the terminal methyl carbon (C9 of DTB).^29−31^

**Figure 9:**
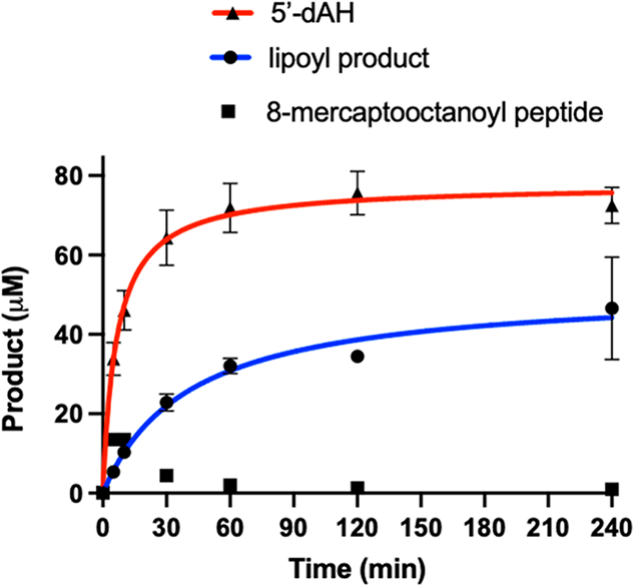
LipS1 and LipS2 together catalyze four turnovers. The reaction contained 10 μM of both LipS1 and LipS2, 300 μM octanoyllysyl peptide substrate, 1 mM dithionite, and 0.5 mM SAM. Reactions were conducted at 45 °C in triplicate. Error bars represent one standard deviation from the mean.

Reactions were also conducted under turnover conditions in the presence of both LipS1 and LipS2. As shown in Figure 9, at 10 μM each enzyme and using **peptide 1** as the substrate, ∼40 μM final lipoyl peptide product and ∼80 μM 5′-dAH are observed. This stoichiometry of 5′-dAH to lipoyl peptide is consistent with the expectation that one 5′-dA· is needed to cleave a C8–H bond, and a second 5′-dA· is needed to cleave a C6–H bond. Under these conditions, LipS1 and LipS2 together catalyze formation of four lipoyl products from the octanoyl peptide substrate. This stoichiometry seems to be limited by the LipS2 reaction, given that ∼40 μM of 8-mercaptooctanoyl product is generated from 10 μM enzyme when using **peptide 1** as a substrate (Figure 7C). By contrast, more than 70 μM product is generated by LipS1 when using **peptide 3** as a substate (Figure 6C). However, when LipS1 and LipS2 are assayed with an in vivo reducing system—ferredoxin, ferredoxin reductase, and NADPH—only 1.5 turnovers are observed for LipS1 and only 1 turnover is observed for LipS2 (Figure S6). These results suggest that the artificial reducing agent dithionite might promote cluster regeneration during in vitro reactions. To address this conundrum, reactions were performed with LipS1 and LipS2 that were produced in their apo forms and then subsequently reconstituted to contain [Fe_4_^34^S_4_] clusters. As shown in Figure S7C,D, reactions conducted in the presence of dithionite afforded substantial amounts of product containing the ^32^S isotope (blue line), indicating that dithionite is a source of additional sulfide in the LipS1 and LipS2 reactions, which is most likely incorporated into the Fe/S clusters of the proteins during turnover. These experiments with isotopically labeled proteins are also consistent with the auxiliary clusters being the source of the appended sulfur atoms.

### Identification of Ligands to the Auxiliary [Fe_4_S_4_] Clusters of LipS1 and LipS2

**Figure 10:**
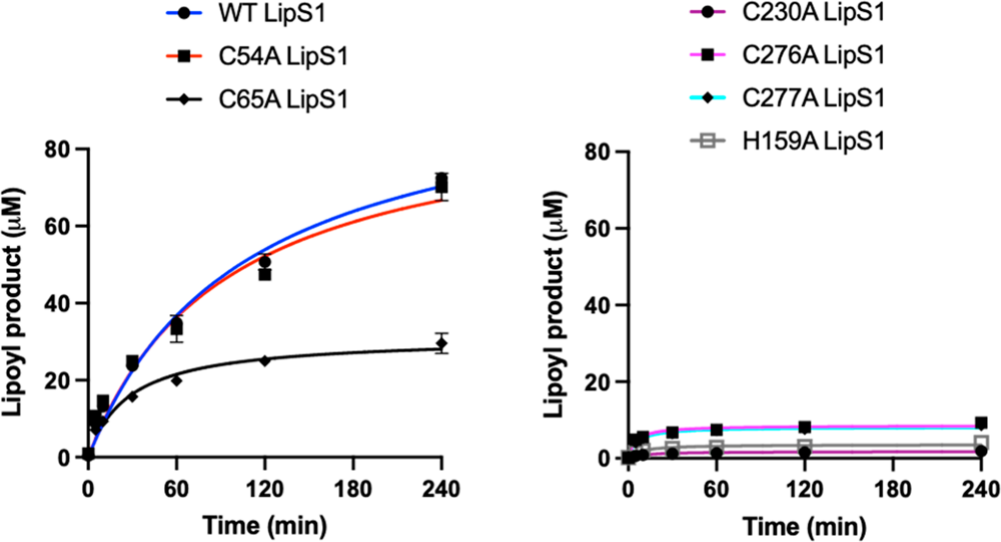
Reactions of wild type and variant LipS1 proteins with the 8-mercaptooctanoyllysyl peptide substrate. WT LipS1 (blue), C54A LipS1 (red), C65A LipS1 (black), C230A LipS1 (maroon), C276A LipS1 (purple), C277A LipS1 (cyan), and H159A LipS1 (gray). Reactions contained 10 μM WT LipS1 or indicated variant, 300 μM 8-mercaptooctanoyllysyl peptide, 0.5 mM SAM, and 1 mM dithionite and were conducted at 45 °C in triplicate. Error bars represent one standard deviation from the mean.

**Figure 11:**
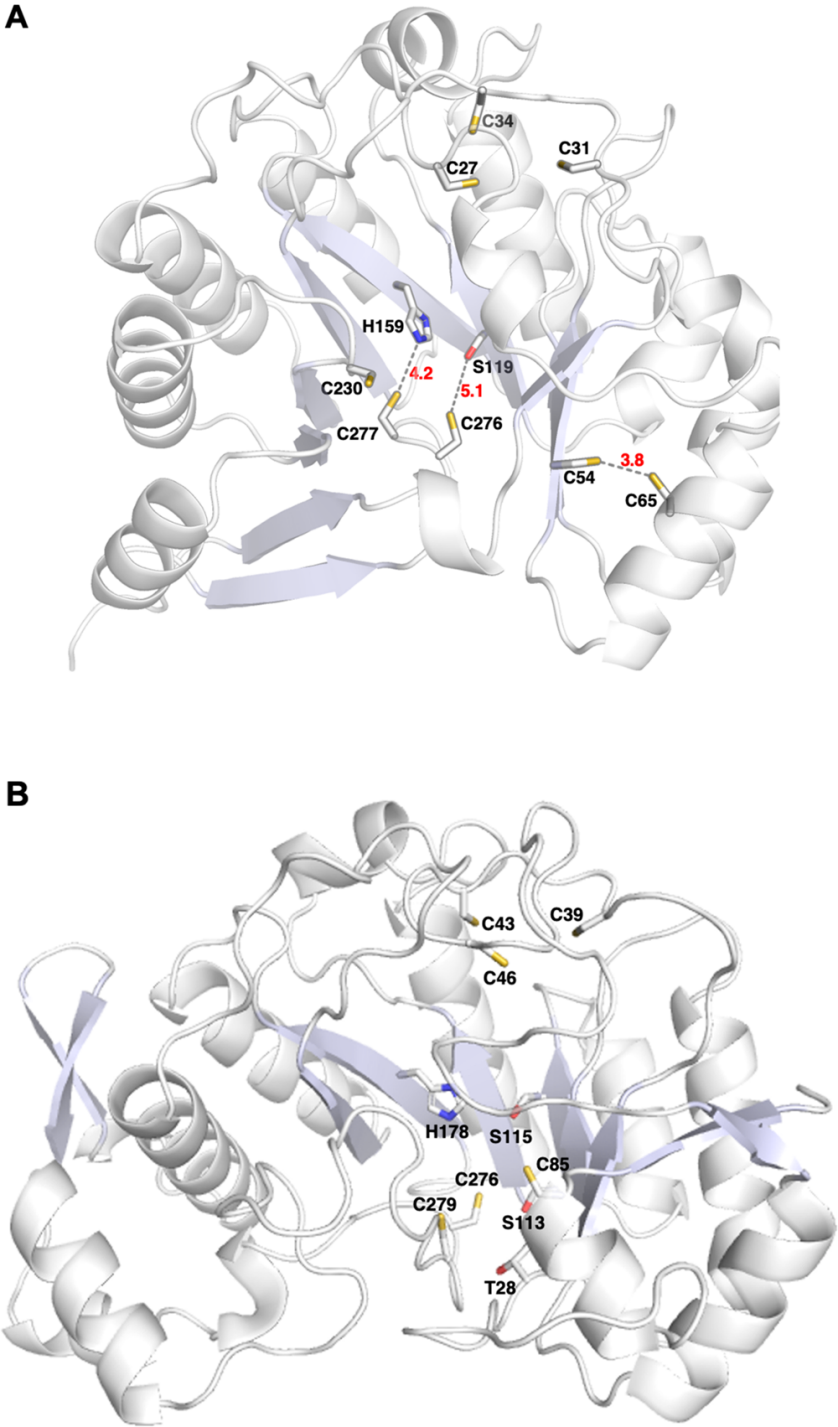
Overall predicted models of (A) LipS1 and (B) LipS2 obtained from AlphaFold. In the LipS1 model, the cysteines in the Cx_3_Cx_2_C motif (C27, C31, C34) that coordinate to the [Fe_4_S_4_]_RS_ cluster and additional cysteines clustered separately at two distinct sites (C54, C65 and C230, C276, C277) are highlighted. In LipS2, the cysteines in the Cx_3_Cx_2_C motif (C39, C43, C46) that coordinate to the [Fe_4_S_4_]_RS_ cluster and additional cysteines that clustered away from the [Fe_4_S_4_]_RS_ cluster (C85, C276, C279) are highlighted.

In addition to LipS1_ΔRS_ and LipS2_ΔRS_, which, as expected, are completely inactive (Figure S8), single Cys → Ala substitutions were made at the remaining cysteines in both proteins to identify which were likely ligands to the [Fe_4_S_4_]_aux_ cluster (Figures S9, S10). The expectation is that each cysteine substitution, if a true ligand, would disrupt the integrity of the auxiliary cluster and inhibit the protein’s ability to catalyze sulfur attachment. LipS1 contains five additional cysteines, while LipS2 contains three (Table 1). The activity of the C54A variant of LipS1 is comparable to that of LipS1_WT_, while that of the C65A variant is moderately reduced. By contrast, the activities of the C230A, C276A, and C277A variants are greatly diminished or undetectable. These results suggest that C230, C276, and C277 are ligands to the LipS1 [Fe_4_S_4_]_aux_ cluster (Figure 10). We generated a model of LipS1 using the AlphaFold server to provide additional evidence for auxiliary cluster ligands (Figure 11A).^63^ Cysteines 27, 31, and 34 reside in the canonical Cx_3_Cx_2_C RS motif and are grouped together in the structure. Accordingly, C230, C276, C277, H159, and S119 are also grouped together in the structure, suggesting that they may be ligands to the auxiliary cluster. The H159A variant is almost completely inactive, while the S119A variant suffered from aggregation and could not be reliably isolated. In the absence of a cluster-bound structure, we are unsure whether H159 or S119 are also ligands; however, a WebLogo of the LipS1 subgroup obtained from RadicalSAM.org indicates that H159 is fully conserved while S119 is not.^20^ In contrast to the aforementioned sets of amino acids, C54 and C65 are located outside of the active site, which is consistent with the observation that Cys → Ala substitutions at these positions result in highly active proteins (Figure 10).

**Table 1:** Radical SAM and Auxiliary Cysteines Present in LipS1 and LipS2

enzyme	cysteines in radical SAM motif (Cx_3_Cx_2_C)	remaining cysteines
LipS1	C^27^x_3_C^31^x_2_C^34^	C^54^, C^65^, C^230^, C^276^, C^277^
LipS2	C^39^x_3_C^43^x_2_C^46^	C^85^, C^276^, C^279^

**Figure 12:**
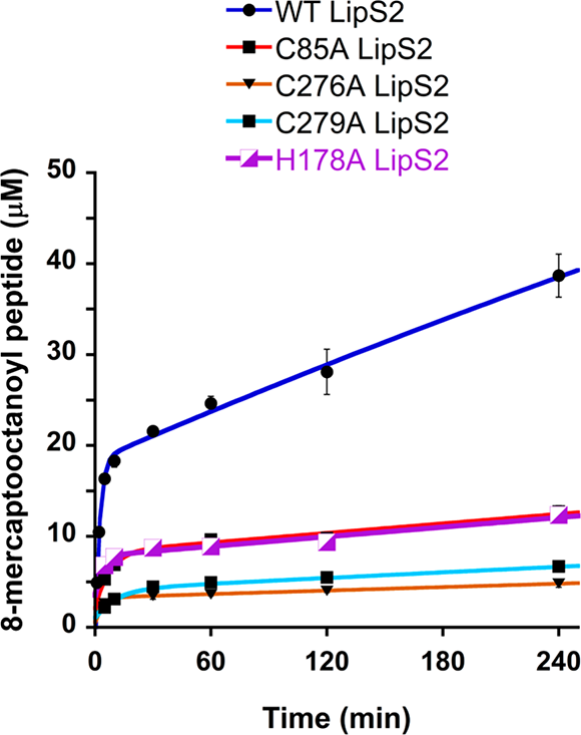
Reactions of wild type LipS2 (blue), C85A LipS2 (red), C276A LipS2 (orange), C279A LipS2 (cyan), and H178A LipS2 (purple) with the octanoyllysyl peptide substrate. The reactions contained 10 μM wt LipS2 or 10 μM indicated variant, 300 μM octanoyllysyl peptide substrate, 0.5 mM SAM, and 1 mM dithionite and were conducted at 45 °C in triplicate. Error bars represent one standard deviation from the mean.

LipS2 contains only three cysteines that are not part of the Cx_3_Cx_2_C motif: C85, C276, and C279. Similar Cys → Ala substitutions were made at these positions, with each of the variant proteins exhibiting significantly diminished activity, although the C85A variant exhibits more activity than the remaining two (Figures 12 and S10). This result suggests that C85, C276, and C279 are ligands to the LipS2 [Fe_4_S_4_]_aux_ cluster, which is supported by the AlphaFold model of LipS2. This model shows that these three cysteines are grouped together in space along with two additional serines (S113 and S115), a threonine (T28), and a histidine (H178) (Figure 11B). Replacement of T28, S113, and S115 residues individually with alanine did not lead to the reduction of the activity of the corresponding LipS2 variant, suggesting that neither of these residues are ligands to the [Fe_4_S_4_]_aux_ cluster (Figure S11A-C). By contrast, replacement of H178 with alanine (H178A) resulted in a significant diminution in activity (Figure 12), suggesting that H178 is likely the fourth ligand to the [Fe_4_S_4_]_aux_ cluster.

## Discussion

Lipoic acid, in the form of the lipoyl cofactor, plays an essential role in aerobic metabolism. The cofactor is typically biosynthesized de novo in a two-step pathway, with the last step being the attachment of two sulfur atoms to an *N*-octanoyllysyl residue on a lipoyl domain-containing protein. Until recently, this last step was believed to be catalyzed uniquely by classical LSs, RS enzymes that contain two essential [Fe_4_S_4_] clusters. One cluster is ligated by cysteines in a Cx_3_Cx_2_C motif and participates in the reductive cleavage of two SAM molecules to yield two 5′-dA·, which are responsible for abstracting H· from C6 and C8 of the substrate to allow for subsequent sulfur attachment. The second cluster is ligated by cysteines in a Cx_4_Cx_5_C motif and is degraded during turnover to supply the attached sulfur atoms. Importantly, the *E. coli* enzyme catalyzes formation of 1 equiv of lipoyl product per enzyme polypeptide in the absence of a system that regenerates the auxiliary cluster after each turnover.

The LS described in this study is composed of two proteins (LipS1, LipS2) and is distinct from classical LSs, although it also uses substrates connected to a lipoyl domain-containing protein rather than free acids. Both LipS1 and LipS2 have the canonical Cx_3_Cx_2_C motif for binding the [Fe_4_S_4_]_RS_ cluster; however, they do not contain the Cx_4_Cx_5_C motif present in classical LSs required for [Fe_4_S_4_]_aux_ cluster coordination. In fact, LipS1 and LipS2 are only 17 and 13% identical to *E. coli* LipA. Unlike *E. coli* LipA, which catalyzes formation of a 6-mercaptooctanoyllysyl intermediate in the first step of catalysis, LipS2 acts on an octanoyllysyl substrate to produce an 8-mercaptooctanoyllysyl product, which is further transformed into the lipoyl product by the action of LipS1. This reaction sequence contrasts with that of classical LSs but is similar to that of *E. coli* BioB, which catalyzes C–S bond formation at the terminal carbon (C9) of dethiobiotin before C–S bond formation at an internal carbon (C6) to afford the thiophane ring of biotin.^29^ Unlike BioB, however, which harbors an [Fe_2_S_2_] auxiliary cluster, our studies indicate that both LipS1 and LipS2, like *E. coli* LipA, harbor [Fe_4_S_4_] auxiliary clusters. LipS1 contains five cysteines in addition to the three that coordinate [Fe_4_S_4_]_RS_ cluster. A model generated using AlphaFold shows that three cysteines (C230, C277, C276) cluster in space, suggesting that they are ligands to the [Fe_4_S_4_]_aux_ cluster. Cys → Ala substitutions at each of these three residues results in proteins that exhibit no significant activity. Similar to the [Fe_4_S_4_]_aux_ cluster in canonical LSs, the unique Fe of the LipS1 [Fe_4_S_4_]_aux_ cluster likely contains a noncysteinyl ligand, which we suggest is the fully conserved H159.^20^ In contrast to LipS1, LipS2 only contains three additional cysteines, suggesting that all three are ligands to the [Fe_4_S_4_]_aux_ cluster of this protein.

A sequence similarity network (SSN) of LipS1 and LipS2 shows that the proteins are found both in archaea and bacteria (Figures S12 and S13). Genome neighborhood network (GNN) analysis indicates that both LipS1 and LipS2 in bacteria cluster with another RS protein as well as with the H protein of the glycine cleavage system and biotin/lipoate A/B protein ligase, all of which are necessary to generate the octanoyllysyl substrate on the H protein and catalyze formation of the lipoyl group. In addition, two proteins that are likely able to catalyze the reversible reduction/oxidation of the dithiolane moiety of the lipoyl group (cysteine-rich domain/heterodisulfide reductase subunit B and heterodisulfide reductase subunit A/pyridine nucleotide-disulfide oxidoreductase) are also found in the neighborhoods of LipS1 and LipS2. In archaea, both LipS1 and LipS2 cluster with another RS protein as well as with the biotin/lipoate A/B protein ligase, while LipS1 clusters additionally with the H protein of the glycine cleavage system. Many archaea that encode canonical LSs also encode the E2 subunit of the PDC, suggesting that the purpose of lipoic acid biosynthesis might be to generate the lipoyl group for occasions when the organism is undergoing aerobic metabolism. By contrast, relatively few archaea that encode LipS1 and LipS2 also encode the E2 subunit of the PDC, raising the question of the role of lipoic acid in these organisms.^44^

## Supporting Information Available

DNA and protein sequences, primers for site-direct mutagenesis, LC-MS activity and multiple-reaction monitoring conditions, Mossbauer spectra and parameters, activity plots, UV–vis spectra, genome neighborhood network analysis (PDF)

## Abbreviations

5′-dA: 5′-deoxyadenosine
DTT: dithiothreitol
EPR: electron paramagnetic resonance
Fe/S: iron sulfur
HEPES: *N*-(2-hydroxyethyl)-piperazine-*N*′-(2-ethanesulfonic acid)
IPTG: isopropyl β-d-1-thiogalactopyranoside
LC-MS/MS: liquid chromatography tandem mass spectrometry
RS: radical SAM
SAH: *S*-adenosylhomocysteine
SAM: *S*-adenosylmethionine
TCEP: tris(2-carboxyethyl)phosphine hydrochloride
Trp: tryptophan
