# Supplementary material for: Characterization of LipS1 and LipS2 from Thermococcus kodakarensis: Proteins Annotated as Biotin Synthases, which Together Catalyze Formation of the Lipoyl Cofactor
Source: ACS Bio Med Chem Au. 2022 Jul 14;2(5):509–20. doi: 10.1021/acsbiomedchemau.2c00018 (PMC9585515; doi:10.1021/acsbiomedchemau.2c00018)
Supplement: Supplementary file 1 — bg2c00018_si_001.pdf [file bg2c00018_si_001.pdf]

Supporting Information for

Characterization of LipS1 and LipS2 from *Theromococcus kodakarensis*: Proteins Annotated as Biotin Synthases, which Together Catalyze Formation of the Lipoyl Cofactor

Syam Sundar Neti[‡], Debangsu Sil[‡], Douglas M. Warui[‡], Olga A. Esakova[‡], Amy E. Solinski[‡], Dante A. Serrano[§], Carsten Krebs[,‡§], and Squire J. Booker*[,‡,§,#]*

[‡]Department of Chemistry, The Pennsylvania State University, University Park, Pennsylvania 16802, USA.

[§]Department of Biochemistry and Molecular Biology, The Pennsylvania State University, University Park, Pennsylvania 16802, USA.

[#]Howard Hughes Medical Institute, The Pennsylvania State University, University Park, Pennsylvania 16802, USA.

*To whom correspondence should be addressed: Squire J. Booker (sjb14@psu.edu); Carsten Krebs (ckrebs@psu.edu)

Table of Contents

1) LipS1 codon optimized gene sequence and protein sequence……….………………….… S3

2) LipS2 codon optimized gene sequence and protein sequence……….…………..……. S4

3) LipS1 auxiliary cysteine mutagenic primers (5′ – 3′)……………..…………..………..S5

4) LipS2 auxiliary cysteine mutagenic primers (5′ – 3′)……………………..………..…..….S6

5) LipS1 & LipS2 RS cysteine mutagenic primers (5′ – 3′)………………………..………S7

6) LC-MS gradient and MRM conditions for product analysis……………………..………S8

7) Non-lipoyl RS enzymes involved in sulfur insertion reactions ………………………………S9

8) EPR spectra of LipS1……………………………………………………………………….S10

9) Mössbauer spectra of reconstituted and as-isolated LipS1$_{\Delta RS}$…..………………………....S11

10) Mössbauer spectra of reconstituted LipS2$_{WT}$………………………………………….....S12

11) Mössbauer spectra of reconstituted LipS2$_{\Delta RS}$……………………………………….....S13

12) Parameters used to simulate Mossbauer spectra……………………………………....S14

13) Activity plots of LipS1$_{WT}$ and LipS2$_{WT}$ using Ferredoxin……………...…………………..S15

14) ^{34}S-labeled LipS1 and LipS2 reactions……………………………………………………..S16

15) Activity plots of LipS1$_{\Delta RS}$ and LipS2$_{\Delta RS}$ variants……………………………………..S17

16) UV-vis spectra of LipS1 and LipS2 auxiliary ligand mutants………………...……………S18

17) Reactions of T28A, S113A, S115A LipS2 with octanoyllysyl peptide……………………S19

18) Genome analysis of LipS1 and LipS2…………………………...……………......S20 – S23

19) UV-vis spectral comparison of LipS1$_{WT}$, LipS1$_{\Delta RS}$……………………..…………...…S24

20) Confidence scores of LipS1 and LipS2 models based on AlphaFold server...……………..S25

LipS1 (TK2109) codon-optimized gene sequence (Uniprot # Q5JEV3)

CATATGGCAGAGCCGAAAAAAAAGCTGAAAATCTATATTCCGGGTATCAAATTTCC
GAGCGTTAGCCTGACCGGTAATGCATGTGCACTGAATTGTGCACATTGCGGTAAACA
TTATCTGGAAGGTATGCGTAAACCGGAACGTGGTGAACTGCTGAGCTATTGTCTGCG
TCTGGCCGAAGAAGGTTATAACGGTTGTCTGCTGAGCGGTGGTATGGATGGTCGTCT
GAAAGTTCCGCTGGATTTTTATGCCAACGAAATCAAAGAGATCAAGAAACGCACCA
ACCTGAAACTGAATGCACATGTGGGTTTTATCGATGAAAGCGATCTGGAATGGGTG
AAATATGTTGATGTTGTGAGCCTGGATTTTGTGGGTGATAATGATGTTATTCGTCGC
GTGTACAAAATCGACAAAACCGTTGATGATTATCTGCGCGTTCTGGATATTCTGACC
GAAGCCGGTGTTCGTGTTGCACCGCATATCACCATTGGTCTGGATTTCGGCAAAATC
CACTGGGAATACAAAGCAATTGATATGCTGGTGAAATACCCGATTGATGTTCTGGTT
CTGGATGTGCTGATTCCGACCAAAGGCACCGAAATGGAAAATGTTCCGAAACCGAG
CGTTGAAGAAAGCCTGGAAGTTGTTAAATATGCCCGTGAAATGTTTGATGGCGAACT
GAGCATTGGTTGTATGCGTCCGTTAGGTCGTTGGCGTCTGGAATTTGATCGTGGTGC
AATTCTGACAGGTGTTGATCGTCTGACCAATCCGCCTCGTAAAGTTATTGAATGGGC
AAAAGGTATTCGTGATGTCGAGATTATCTATGAATGCTGCGTGATGTAACTCGAG

CATATG – *NdeI* restriction site
CTCGAG – *XhoI* restriction site
TAA – Stop codon

LipS1 (TK2109) protein sequence

MGSSHHHHHHSSGLVPRGSHMAEPKKKLKIYIPGIKFPSVSLTGNACALNCAHCGKHYL
EGMRKPERGELLSYCLRLAEEGYNGCLLSGGMDGRLKVPLDFYANEIKEIKKRTNLKLN
AHVGFIDESDLEWVKYVDVVSLDFVGDNDVIRRVYKIDKTVDDYLRVLDILTEAGVRV
APHITIGLDFGKIHWEYKAIDMLVKYPIDVLVLDVLIPTKGTEMENVPKPSVEESLEVVK
YAREMFDGELSIGCMRPLGRWRLEFDRGAILTGVDRLTNPPRKVIEWAKGIRDVEIIYEC
CVM

LipS2 (TK2248) codon-optimized gene sequence (Uniprot # Q5JHS9)

CATATGCCGGAAATGGTTCGTGTTAGCTATGGCACCGCAATTGCAATGGGTCTGATT
CGTGCAAAACTGCTGGCACGTCCGACCACCGCATATCTGATGACCTATTGGCCTGGT
CGTTGTAGCAATGATTGTGCATTTTGTGCACAGGCACGTAGCAGCCGTGCAGATCTG
GAAAAACTGAGCCGTGTTGTTTGGCCGAGCTTTATGCTGGAAGATGTTCTGGAAGGT
CTGAAAAAAGGTAACTTTGCACGTATTTGCCTGCAGACCATTGATTATCCTGGTATG
GTTGAAGATGTGTTCGATCTGCTGGAAGCATTTAGCGATCTGAATCTGCCGATTAGC
GTTAGCATTACACCGGTTGATAGCGAAACCCTGGAACGTTTTAAAGAACGTGGTGTG
GATTATATTGGTGTTGGTCTGGATGTTGCAAGCGAACGTCTGTTTCGTGAAATTAAA
CCGGATCTGAGCTGGGAAGAAGTTTGGGATTTTGCAGGTCGTGTTATTGATGTTTTT
GGTCGTGGTAAAGCACTGCTGCATGTTATTGTTGGCCTGGGTGAAACCGATGGTGAA
CTGGTTAATACCTTTATTCGTGCACGTGAAATTGGTGCCGATGTTAGCATTTTTGCAT
TTACCCCGATTAAAGGCACCCGTCTGGAAAATCGTGAACCGCCTAGCCTGGAACATT
ATCGTAAAATTCAGCTGGCCAAATATCTGGTGAGCATTGGTAAAGAAGATGCCATTG
TTTTTGATGGCGATAGCATTAAAGGTTTCGCACTGAGCAAAGATGAAGTTGCAAAAA
TTCCGACCACAGTGTTTATGACCCATGGTTGTCCGGGTTGTAATCGTCCGTATTATAA
CGAACGTCCGGGTAAAGAACCGTATAACTATCCGCTGGCACCGAAACGTGAAGATT
TTGAACGTACCCTGCGTCTGATTCTGTAACTCGAG

CATATG – *NdeI* restriction site
CTCGAG – *XhoI* restriction site
TAA – Stop codon

LipS2 (TK2248) protein sequence

MGSSHHHHHHSSGLVPRGSHMPEMVRVSYGTAIAMGLIRAKLLARPTTAYLMTYWPG
RCSNDCAFCAQARSSRADLEKLSRVVWPSFMLEDVLEGLKKGNFARICLQTIDYPGMV
EDVFDLLEAFSDLNLPISVSITPVDSETLERFKERGVDYIGVGLDVASERLFREIKPDLSW
EEVWDFAGRVIDVFGRGKALLHVIVGLGETDGELVNTFIRAREIGADVSIFAFTPIKGTRL
ENREPPSLEHYRKIQLAKYLVSIGKEDAIVFDGDSIKGFALSKDEVAKIPTTVFMTHGCPG
CNRPYYNERPGKEPYNYPLAPKREDFERTLRLIL

Table S1: Primers for constructing LipS1 auxiliary cysteine and other ligand variants

Mutant	Forward primer	Reverse primer
C54A LipS1	5'-GAACTGCTGAGCTATGCGC TGCGTCTGGCCGAA-3'	5'-CTTCGGCCAGACGCAGCG CATAGCTCAGCAGTTC-3'
C65A LipS1	5'-GAAGAAGGTTATAACGGTGC GCTGCTGAGCGGTGGTATG-3'	5'-CATACCACCGCTCAGCAGCG CACCGTTATAACCTTCTTC-3'
C230A LipS1	5'-GGCGAACTGAGCATTGGTGC GATGCGTCCGTTAGGTCG-3'	5'-CGACCTAACGGACGCATCGC ACCAATGCTCAGTTCGCC-3'
C276A LipS1	5'-GTCGAGATTATCTATGAAGC GTGCGTGATGTAACTCGAG-3'	5'-CTCGAGTTACATCACGCACG CTTCATAGATAATCTCGAC-3'
C277A LipS1	5'-GTCGAGATTATCTATGAATG CGCGGTGATGTAACTCGAG-3'	5'-CTCGAGTTACATCACCGCGC ATTCATAGATAATCTCGAC-3'
H159A LipS1	5'- GGTGTTCGTGTTGCACCG GCGATCACCATTGGTCTG -3'	5'-CAGACCAATGGTGATCGCCG GTGCAACACGAACACC-3'

Table S2: Primers for constructing LipS2 auxiliary cysteine and other ligand variants

Mutant	Forward primer	Reverse primer
C85A LipS2	5’-GGTAACTTTGCACGTATTGCG<br>CTGCAGACCATTGATTATCCTG-3’	5’-CAGGATAATCAATGGTCTGCA<br>GCGCAATACGTGCAAAGTTACC-3’
C276A LipS2	5’-GTGTTTATGACCCATGGTGCG<br>CCGGGTTGTAATCGTCCG-3’	5’-CGGACGATTACAACCCGGC<br>GCACCATGGGTCATAAACAC-3’
C279A LipS2	5’-GACCCATGGTTGTCCGGGT<br>GCGAATCGTCCGTATTATAACG-3’	5’-CGTTATAATACGGACGATTC<br>GCACCCGGACAACCATGGGTC-3’
T28A LipS2	5’-GCTGGCACGTCCGACCGCG<br>GCATATCTGATGACCTATTG-3’	5’-CAATAGGTCATCAGATATGC<br>CGCGGTCGGACGTGCCAGC-3’
S113A LiPS2	5’-GATCTGAATCTGCCGATT<br>GCGGTTAGCATTACACCG-3’	5’-CGGTGTAATGCTAACCGCAA<br>TCGGCAGATTCAGATC-3’
S115A LipS2	5’-GAATCTGCCGATTAGCGTT<br>GCGATTACACCGGTTGATAG-3’	5’-CTATCAACCGGTGTAATCG<br>CAACGCTAATCGGCAGATTC-3’
H178A LipS2	5’-GTCGTGGTAAAGCACTGCTG<br>GCGGTTATTGTTGGCCTGGG-3’	5' -CCCAGGCCAACAATAACGC<br>CAGCAGTGCTTTACCACGAC-3’

Table S3: Primers for constructing LipS1$_{\Delta RS}$ and LipS2$_{\Delta RS}$ cysteine variants

Mutant	Forward primer	Reverse primer
LipS1 C27A/C31A/ C34A	5'-GCCTGACCGGTAATGCAGCGGCACTGAATGCGGCACATGCGGGTAAACATTATCTG-3'	5'-CAGATAATGTTTACCCGCATGTGCCGCATTCAGTGCCGCTGCATTACCGGTCAGGC-3'
LipS2 C39A/C43A/ C46A	5'-GACCTATTGGCCTGGTCGTGCGAGCAATGATGCGGCATTTGCGGCACAGGCACGTAGCAG-3'	5'-CTGCTACGTGCCTGTGCCGCAAATGCCGCATCATTGCTCGCACGACCAGGCCAATAGGTC-3'

Table S4: LC-MS gradient conditions for the analysis of substrates/products in LipS1 and LipS2 reactions

Time (min)	0.1% Formic acid in H_2O	Acetonitrile	Flow (mL/min)
0	95%	5%	0.3
0.5	95%	5%	0.3
3	50%	50%	0.3
4.5	50%	50%	0.3
5.5	95%	5%	0.3
7	95%	5%	0.3

Table S5: MRM fragmentation products monitored by LC-MS.

Compound	Parent ion*	Product Ion 1§	Product 1on 2§
AtsA peptide	474.4 (112)	229.1 (18)	153 (26)
5′-dAH	252.1 (90)	136 (13)	119 (50)
Trp	188 (114)	145.9 (13)	117.9 (25)
Methionine	150.1 (65)	104 (8)	56 (16)
Lipoyl peptide	996.5 (208)	776.3 (30)	274.1 (46)
Monothiolated peptide	964.5 (222)	584.3 (32)	242.1 (48)
Octanoyl peptide	932.5 (188)	712.4 (29)	210.1 (53)
Monothiolated ^{34}S peptide	966.5 (222)	586.3 (32)	244.1 (48)
Lipoyl $^{34/32}$S peptide	998.5 (208)	778.3 (30)	276.1 (46)

*Respective fragmentor voltage in parenthesis
§ Respective collision energies in parenthesis

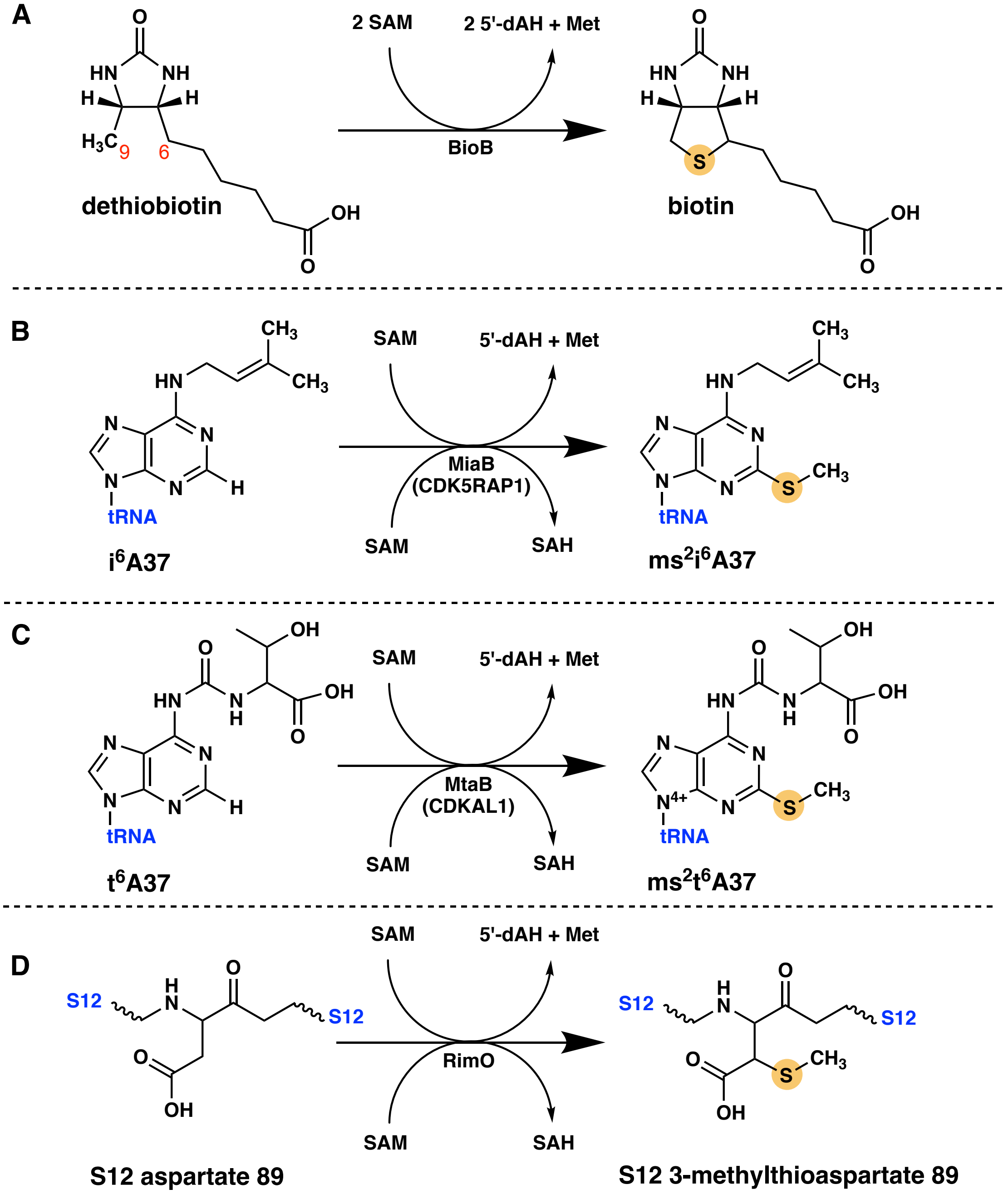

Figure S1: Other RS enzymes involved in sulfur insertion reactions **A)** BioB **B)** MiaB **C)** MtaB **D)** RimO

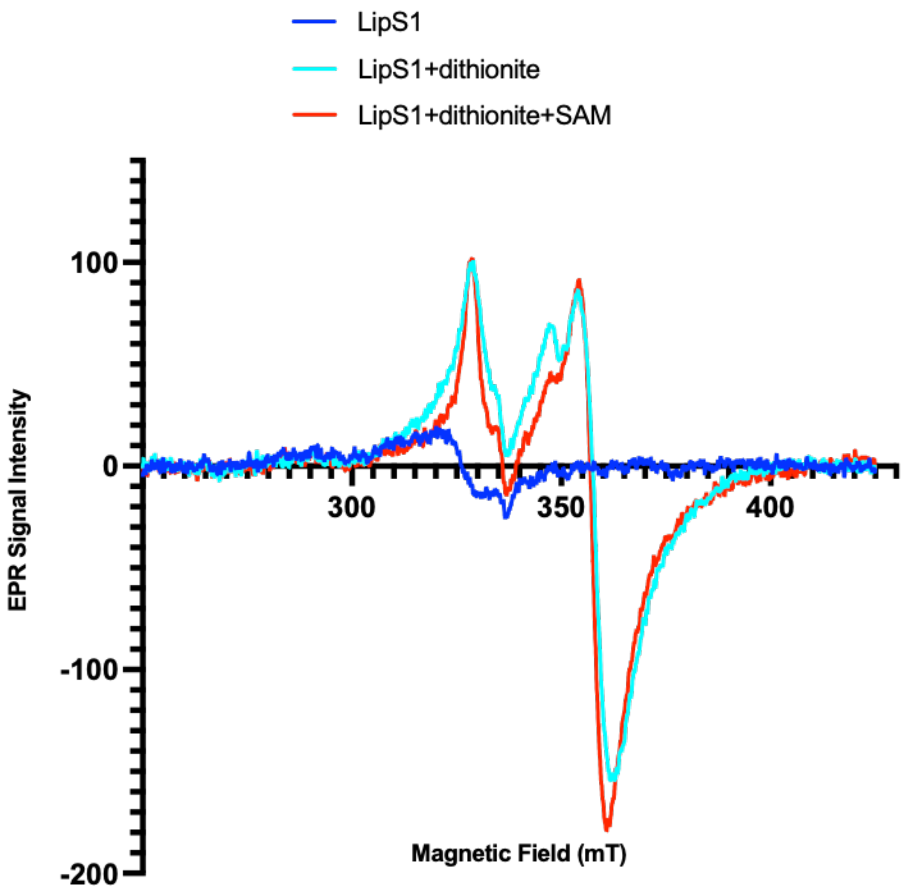

Figure S2. EPR spectra of 200 μM LipS1$_{WT}$ (blue), 200 μM LipS1$_{WT}$ + 2 mM dithionite (cyan), and 200 μM LipS1$_{WT}$ + 2 mM dithionite + 1 mM SAM (red). Spectra were recorded at 10 K.

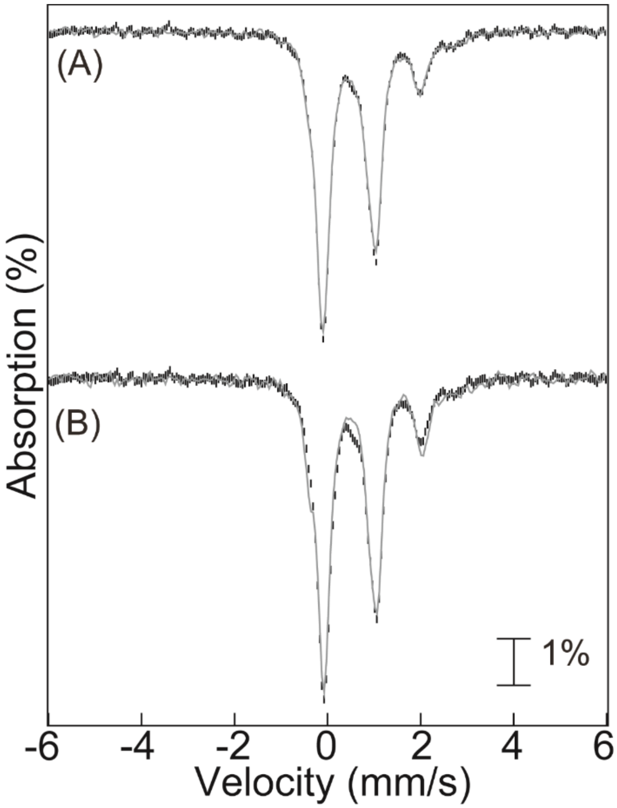

Figure S3. Comparison of the 4.2-K Mössbauer spectrum of reconstituted LipS1$_{\Delta RS}$ collected without an externally applied magnetic field (vertical bars) to the 4.2-K Mössbauer spectrum of reconstituted LipS1$_{\Delta RS}$ collected with a 53-mT magnetic field oriented parallel to the γ beam (solid line, **A**); the fact that these spectra are virtually identical reveals the absence of $[Fe_3S_4]^0$-like clusters. Comparison of the 4.2-K Mössbauer spectrum of reconstituted LipS1$_{\Delta RS}$ collected without an externally applied magnetic field (vertical bars) to the 4.2-K Mössbauer spectrum of non-reconstituted LipS1$_{\Delta RS}$ collected without an externally applied magnetic field (solid line, **B**). The fact that the major contribution to the spectra is the quadrupole doublet associated with $[Fe_4S_4]^{2+}$ clusters suggests that the auxiliary cluster as is an $[Fe_4S_4]$ cluster.

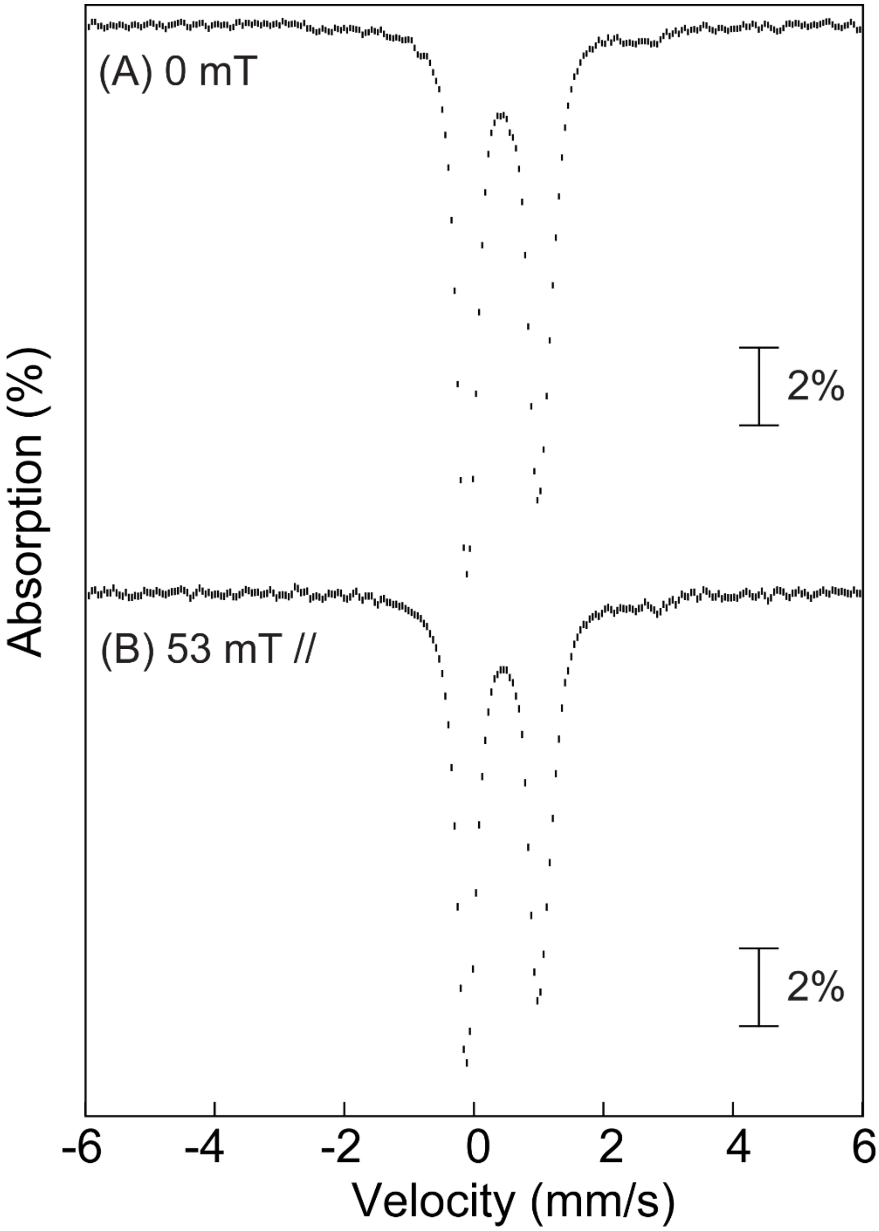

Figure S4. Mössbauer spectra of reconstituted LipS2$_{WT}$ collected at 4.2 K with an external applied magnetic field of (A) 0 mT or (B) 53 mT oriented parallel to the γ beam (vertical bars).

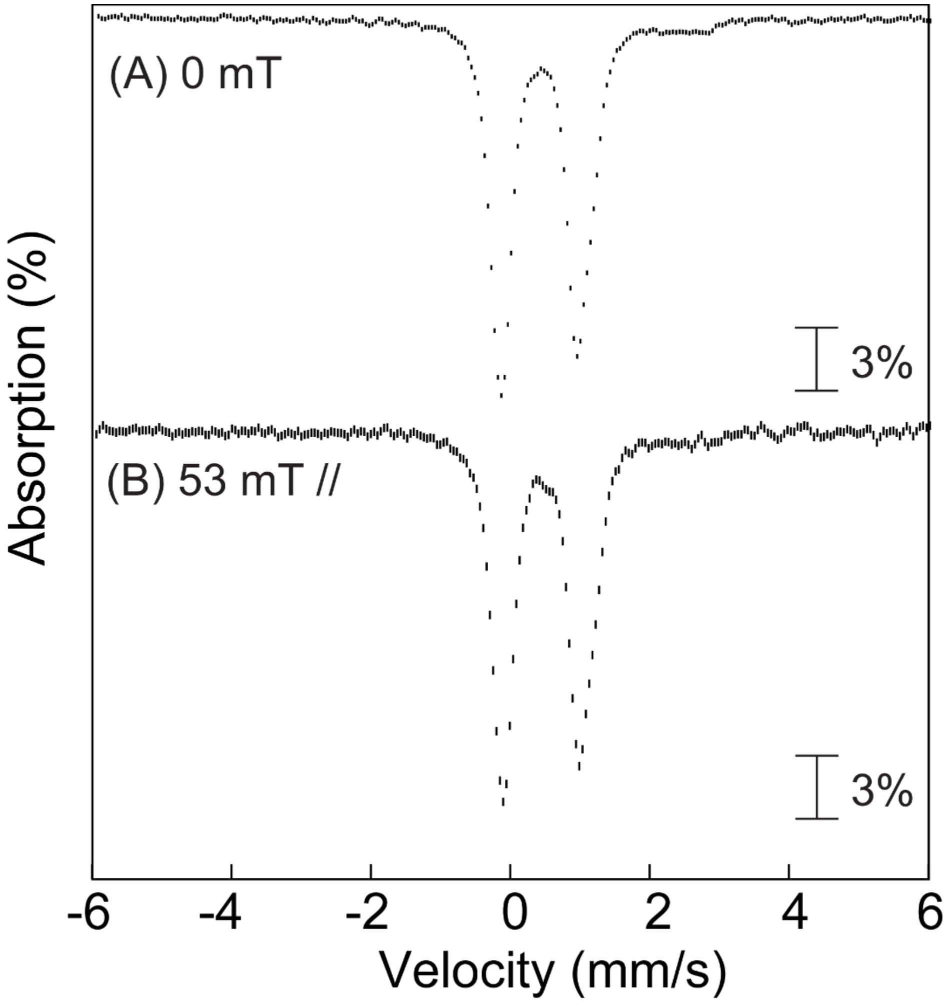

Figure S5. Mössbauer spectra of reconstituted LipS2$_{\Delta RS}$ collected at 4.2 K with an external applied magnetic field of (A) 0 mT or (B) 53 mT oriented parallel to the γ beam (vertical bars).

Table S6: Parameters used to simulate Mössbauer spectra

Protein	Fe species		δ (mm/s)	ΔE_Q (mm/s)	% Total ^{57}Fe	^{57}Fe/protomer	# of cluster(s)/ protomer
LipS1$_{WT}$	$[Fe_4S_4]^{2+}$		0.46	1.12	47	3.29	0.8
	[3Fe-4S]^0	$Fe_2^{2.5}$	0.45	1.00	20	2.10	0.7
		Fe^{III}	0.31	0.50	10		
	$[Fe_2S_2]^{2+}$		0.25	0.67	12	0.84	0.4
	N/O-coordinated high-spin Fe^{II}		1.12	2.67	11	0.77	-
LipS1$_{ΔRS}$	$[Fe_4S_4]^{2+}$		0.46	1.12	69	2.83	0.7
	N/O-coordinated unique Fe site of $[Fe_4S_4]^{2+}$		0.85	2.25	17	0.70	0.3
	$[Fe_2S_2]^{2+}$		0.25	0.67	9	0.37	0.2
	N/O-coordinated high-spin Fe^{II}		1.20	2.9	5	0.2	-
LipS2$_{WT}$	$[Fe_4S_4]^{2+}$		0.47	1.12	90	6.12	1.5
	$[Fe_2S_2]^{2+}$		0.25	0.67	5	0.34	0.2
	N/O-coordinated high-spin Fe^{II}		1.28	2.71	5	0.34	-
LipS2$_{ΔRS}$	$[Fe_4S_4]^{2+}$		0.47	1.12	90	3.48	0.9
	$[Fe_2S_2]^{2+}$		0.25	0.67	2	0.08	0.04
	N/O-coordinated high-spin Fe^{II}		1.28	2.71	8	0.31	-

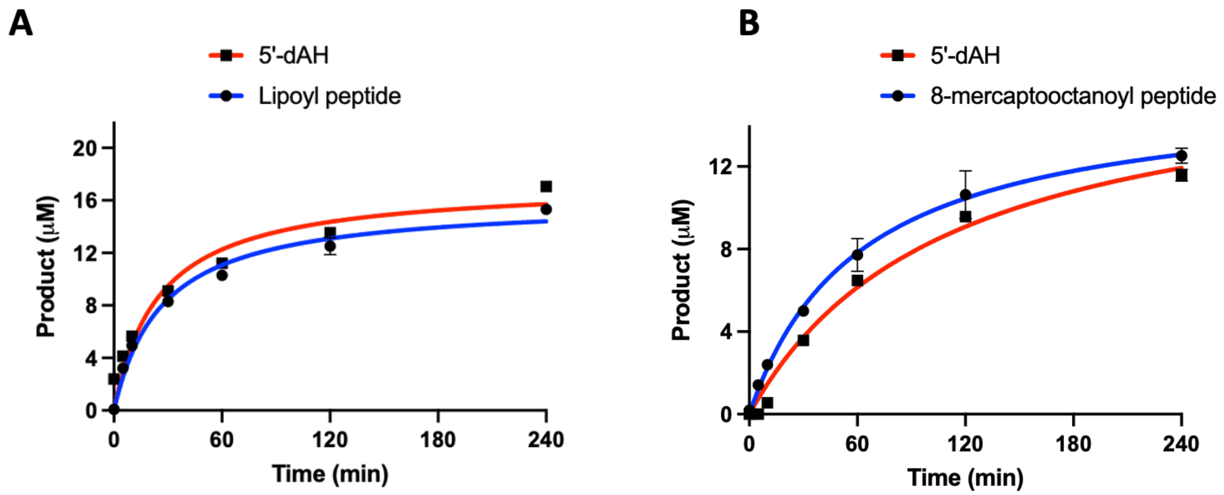

Figure S6. Reactions of **A)** LipS1$_{WT}$ with 8-mercaptooctanoyl peptide substrate **B)** LipS2$_{WT}$ with octanoyl peptide substrate. The reactions contained 10 μM LipS1 or 10 μM LipS2, 300 μM peptide substrate, 10 μM ferredoxin, 4 μM ferredoxin reductase, 1 mM NADPH and 0.5 mM SAM. Reactions were conducted at 40 ^{o}C in triplicate. Error bars represent one standard deviation from the mean.

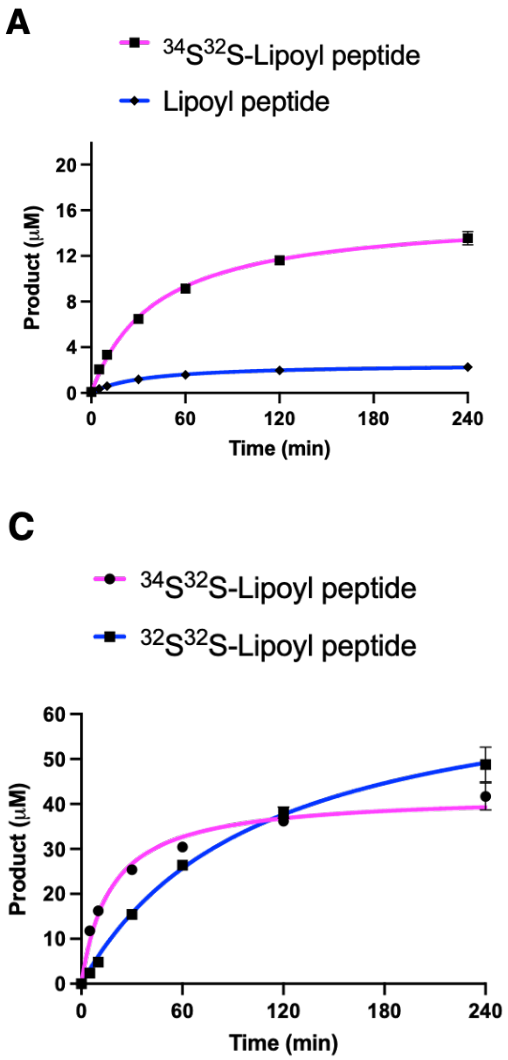

Figure S7. A) and **B)** ^{34}S-labeled LipS1 and ^{34}S-labeled LipS2 reactions with 8-mercaptooctanoyllysyl peptide or with octanoyllysyl peptide, respectively, using the ferredoxin/ferredoxin reductase/NADPH reducing system. **C)** and **D)** ^{34}S-labeled LipS1 and ^{34}S-labeled LipS2 reactions with 8-mercaptooctanoyllysyl peptide and octanoyllysyl peptide, respectively, using dithionite as a reductant. The reactions contained 10 µM LipS1 or 10 µM LipS2, 10 µM ferredoxin, 4 µM ferredoxin reductase, and 1.5 mM NADPH, 300 µM substrate, and 0.5 mM SAM. When dithionite replaced the ferredoxin/ferredoxin reductase/NADPH

reducing system it was used at a concentration of 1.5 mM. Reactions were conducted at 40 °C in triplicate. Error bars represent one standard deviation from the mean.

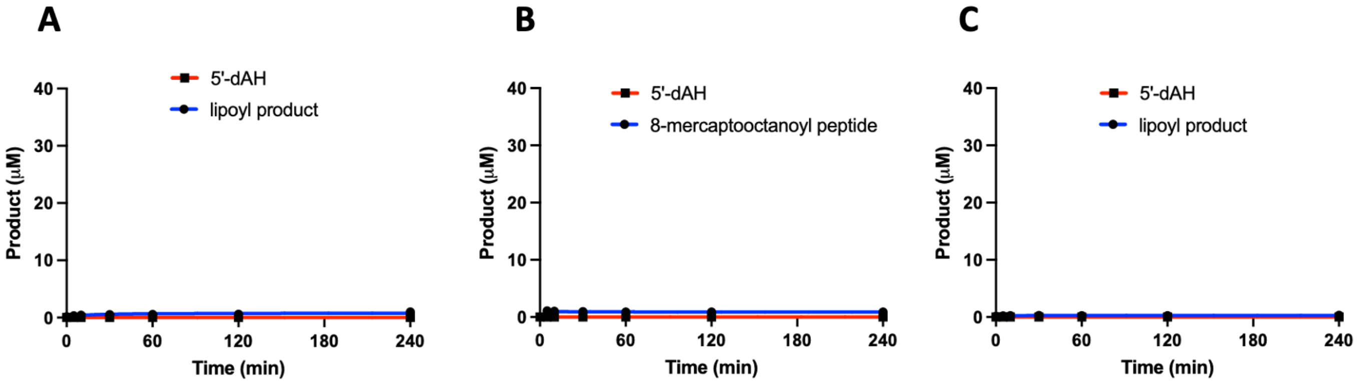

Figure S8. Reactions of **A)** LipS1$_{\Delta RS}$ with 8-mercaptooctanoyl peptide substrate **B)** LipS2$_{\Delta RS}$ with octanoyl peptide substrate **C)** LipS1$_{\Delta RS}$ and LipS2$_{\Delta RS}$ with octanoyl peptide. The reactions contained 10 μM LipS1 and/or 10 μM LipS2, 300 μM octanoyl peptide substrate, 0.5 mM dithionite and 0.5 mM SAM. Reactions were conducted at 45 °C in triplicate. Error bars represent one standard deviation from the mean.

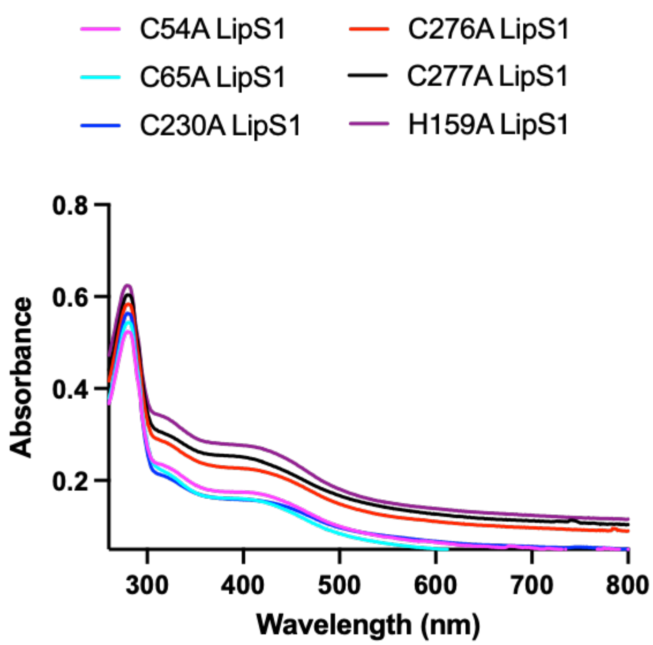

Figure S9: UV-Vis spectra of 8.4 μM LipS1 auxiliary cluster ligand variants (normalized to the absorption at 280 nm).

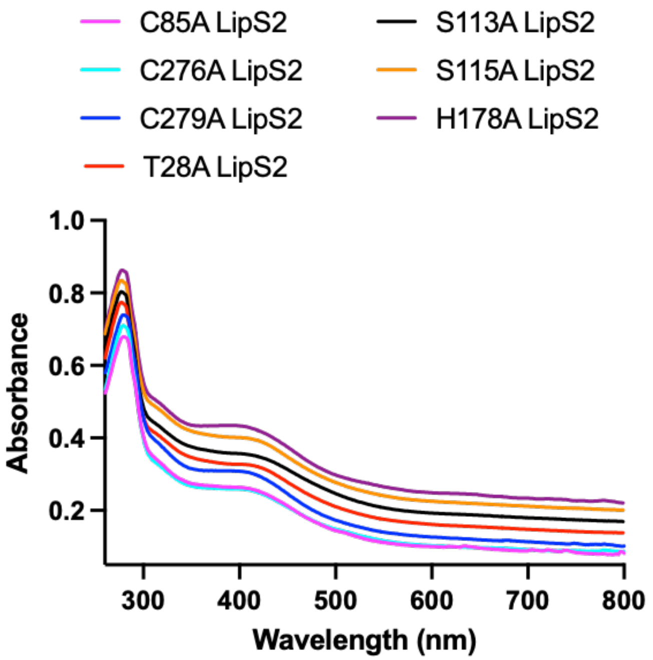

Figure S10: UV-Vis spectra of 9 μM LipS2 auxiliary cluster ligand variants (normalized to the absorption at 280 nm).

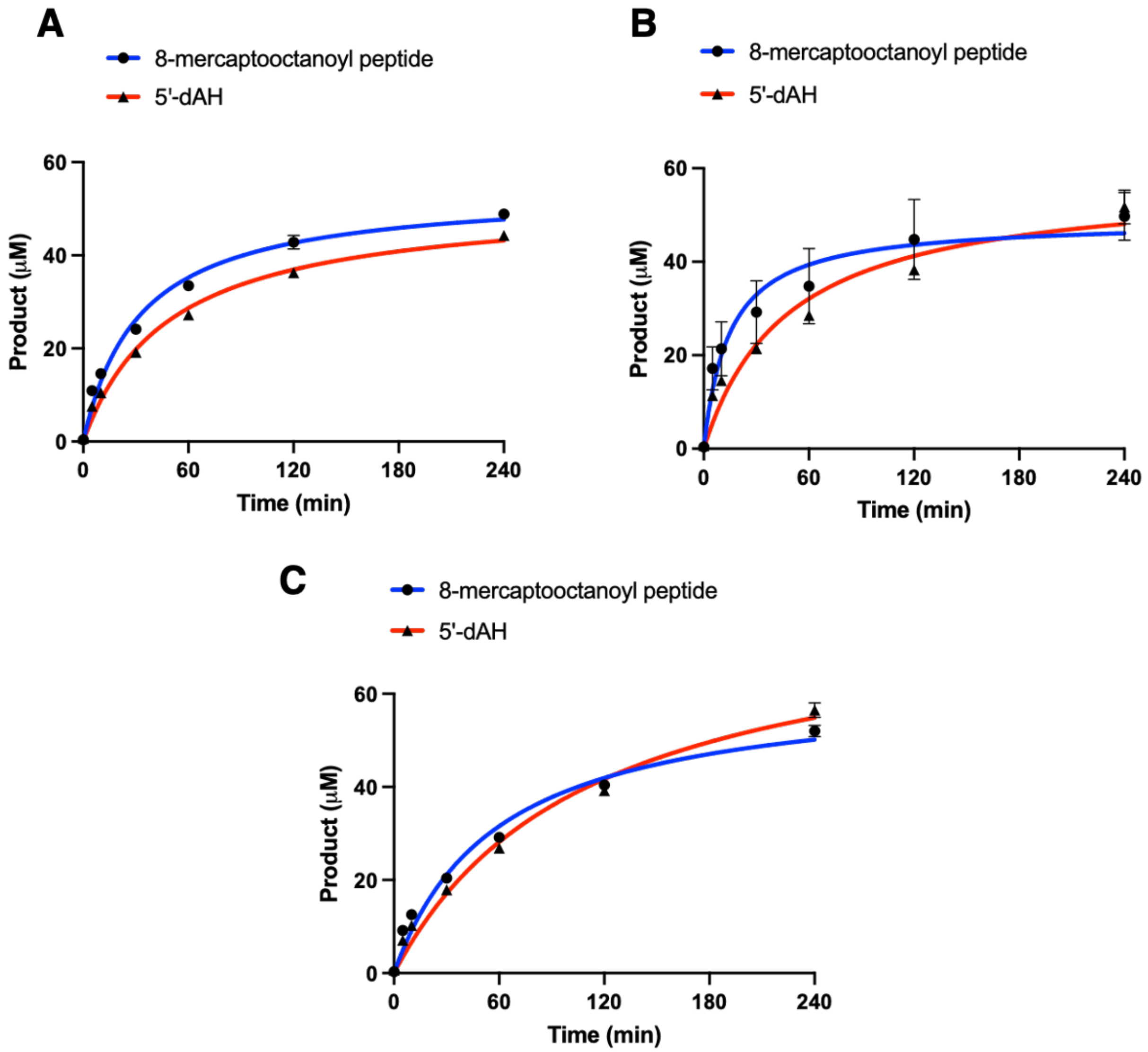

Figure S11. Reactions of **A)** T28A LipS2 **B)** S113A LipS2 **C)** S115A LipS2 with octanoyllysyl peptide. The reactions contained 10 μM LipS2 variant, 300 μM substrate, 1.5 mM dithionite and 0.5 mM SAM. Reactions were conducted at 45 ^{o}C in triplicate. Error bars represent one standard deviation from the mean.

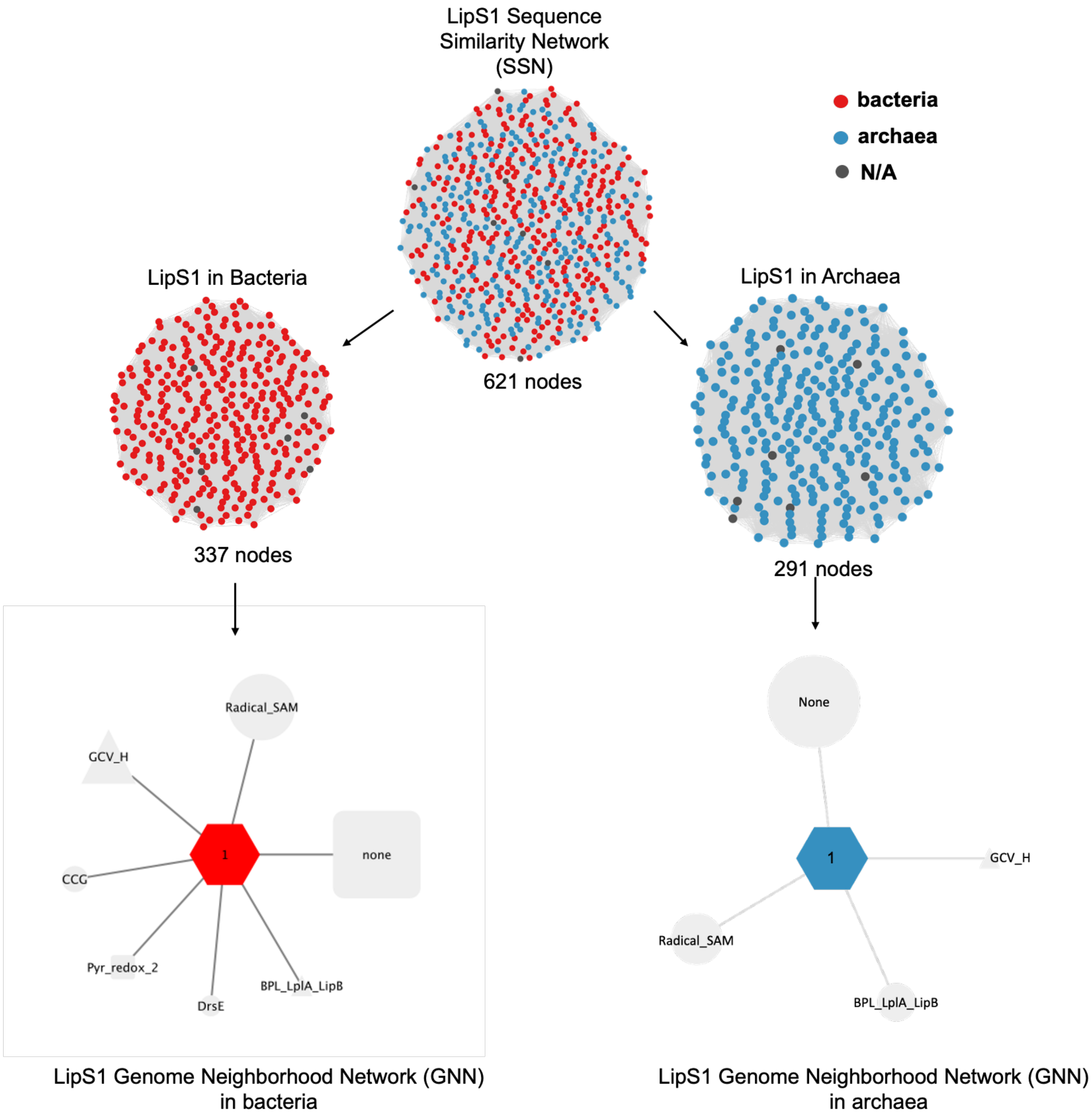

Figure S12. Genome neighborhood analysis of Megacluster 4_7 (LipS1) in bacteria and archaea obtained from radicalSAM.org.

GCV_H = H-protein of the glycine cleavage system (Pfam01597)

Radical_SAM = Radical-SAM domain containing protein (Pfam04055)

CCG = cysteine-rich domain/heterodisulfide reductase subunit B (Pfam02754)

BPL_LplA_LipB = Biotin/lipoate A/B protein ligase family (Pfam03099)

Pyr_redox_2 = Heterodisulfide reductase subunit A/pyridine nucleotide-disulphide oxidoreductase (Pfam07992)

DrsE = Putative peroxiredoxin/sulfur reduction protein (Pfam02635)

None = Neighbor not associated with a Pfam family (20% of protein in UniProt are not assigned to a Pfam family). 'None' assigned to the 'no Pfam' family.

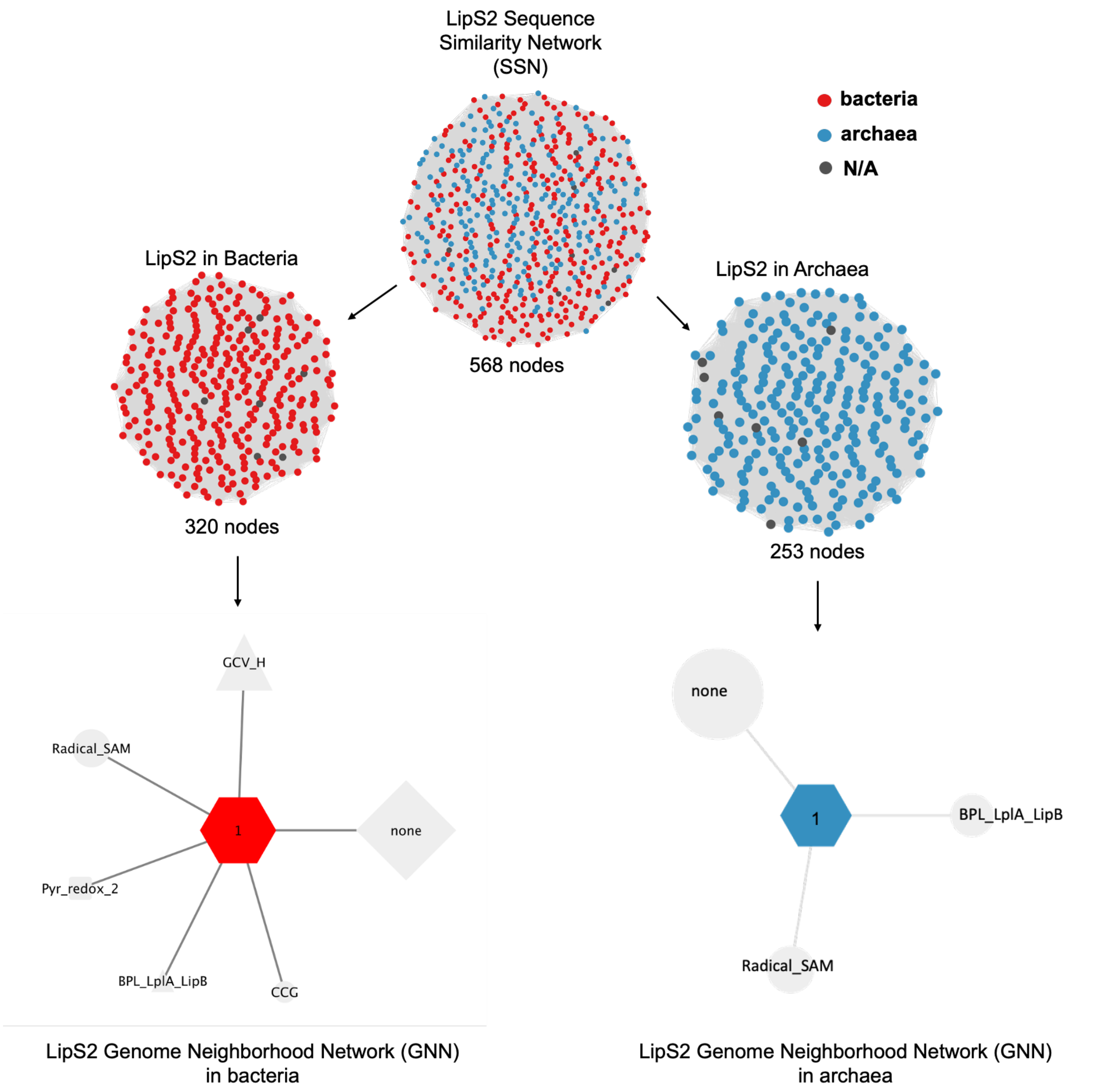

Figure S13. Genome analysis of Megacluster 4_8 (LipS2) in bacteria and archaea obtained from radicalSAM.org.

GCV_H = H-protein of the glycine cleavage system (Pfam01597)

Radical_SAM = Radical-SAM domain containing protein (Pfam04055)

CCG = cysteine-rich domain/heterodisulfide reductase subunit B (Pfam02754)

BPL_LplA_LipB = Biotin/lipoate A/B protein ligase family (Pfam03099)

Pyr_redox_2 = Heterodisulfide reductase subunit A/pyridine nucleotide-disulphide oxidoreductase (Pfam07992)

None = Neighbor not associated with a Pfam family (20% of protein in UniProt are not assigned to a Pfam family). 'None' assigned to the 'no Pfam' family.

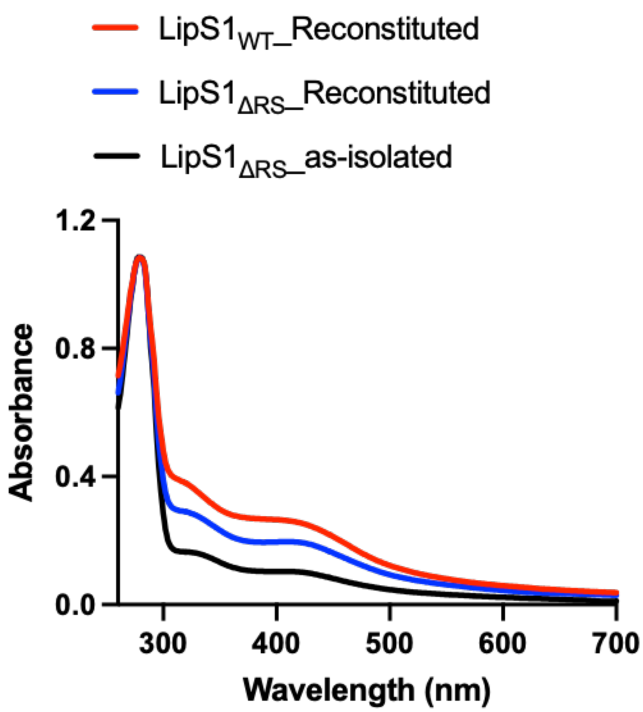

Figure S14. Comparison of the UV-vis spectra of reconstituted LipS1$_{WT}$ (red), reconstituted LipS1$_{\Delta RS}$ (blue), and as-isolated LipS1$_{\Delta RS}$ (black) proteins (normalized to the absorption at 280 nm).

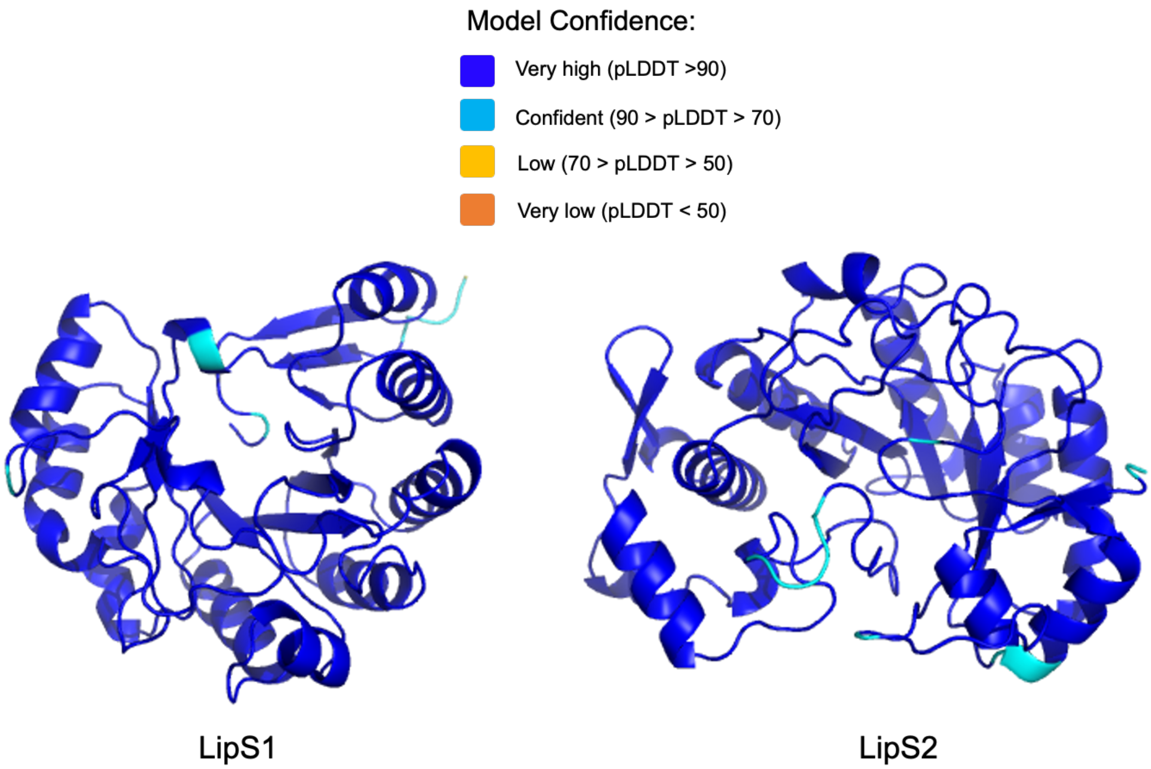

Figure S15. Confidence scores of LipS1 and LipS2 models based on AlphFold server. AlphaFold server predicts per-residue confidence score (pLDDT) in the range of 0 to 100.
